# Supplementary material for: Completion of isoniazid–rifapentine (3HP) for tuberculosis prevention among people living with HIV: Interim analysis of a hybrid type 3 effectiveness–implementation randomized trial
Source: PLoS Med. 2021 Dec 16;18(12):e1003875. doi: 10.1371/journal.pmed.1003875 (PMC8726462; doi:10.1371/journal.pmed.1003875)
Supplement: S1 Statistical Analysis Plan — (DOCX) [file pmed.1003875.s004.docx]

**3HP Options Trial: Interim Analysis Statistical Analysis Plan**

**Trial Background**

1. **Trial summary:** The *3HP Options Trial* is a pragmatic randomized trial to compare three optimized strategies for delivering tuberculosis preventive therapy (TPT) to people living with HIV (PLHIV) using the short-course 3HP regimen. Eligible participants are randomized to one of three arms to receive 3HP treatment with once weekly isoniazid (INH) and rifapentine (RPT) for 12 weeks given by either facilitated directly observed therapy (DOT), facilitated self-administered therapy (SAT), or an informed choice between facilitated DOT and facilitated SAT (with the assistance of a decision aid tool).
2. **Trial objective:** To compare the uptake of 3HP under three delivery strategies: 1) Facilitated DOT; 2) Facilitated SAT; and 3) Informed patient choice (using a decision aid) between facilitated DOT and facilitated SAT.

### Trial Primary Outcome (Effectiveness of delivery options):

| **Outcome** | **Numerator** | **Denominator** |
| --- | --- | --- |
| *Proportion accepting and completing 3HP treatment* | *Number who take at least 11 of 12 doses within 16 weeks from date of enrollment* | *Number randomized* |

1. **Trial co-primary hypothesis**
   1. The proportion of PLHIV who accept and complete 3HP can exceed 80% in a high HIV/TB burden setting.

**Interim Analysis Methods**

1. **Interim Analysis Outcomes:** Proposed outcomes for the interim analysis are displayed in the Table below and include the proportion who accept 3HP treatment and complete at least 11 of the 12 weekly doses within 16 weeks of enrollment, and the proportion who had a serious adverse event (AE) resulting in treatment discontinuation.

| **Outcome** | **Numerator** | **Denominator** |
| --- | --- | --- |
| 1. *Proportion accepting and completing 3HP treatment** | *Number who took at least 11 of 12 doses within 16 weeks from date of enrollment* | *Number no longer on treatment among those randomized* |
| 1. *Proportion who had a serious adverse event (AE)* | *Number with a serious AE that resulted in treatment discontinuation* | *Number no longer on treatment among those initiating treatment* |

*Primary outcome

1. **Statistical Analysis**:
2. Summarize patient clinical and sociodemographic characteristics using numbers and percentages, and mean values with standard deviation.
3. Calculate and report the outcome proportion (and exact binomial 95% confidence interval) of patients accepting and completing 3HP treatment among those randomized, aggregated across all study arms (facilitated DOT, facilitated SAT and patient choice). We will present this outcome proportion overall and also stratified by sex, age category (18-41 years vs. ≥42 years), and time on ART (≤1 year vs. >1 year).
4. Calculate and report the proportion of patients who discontinued 3HP due to a serious AE among those initiating treatment, aggregated across all study arms.
5. Use Bayesian inference analysis to estimate the posterior probability that the proportion of PLHIV who accept and complete 3HP, denoted here as *θ,* exceeds 0.8 in at least one of the three study arms*.* To be conservative, we will assume that the outcomes are distributed equally across the three study arms, namely that 148 of 160 (93%) individuals have completed 3HP in each arm.

| **Patients exiting by date (actual)** | **Outcome proportion** | **Sample size (Total/By Arm)** | **Number getting primary outcome/arm** | **Parameter** |
| --- | --- | --- | --- | --- |
| 4/30/21 | 0.93 | 160 | 148 | 0.93 |

As the outcome is a binary response variable (achieved the primary outcome vs. did not achieve the primary outcome), we will use a beta distribution to the outcome parameter *θ.* We will use a non-informative flat conjugate beta(1,1) prior, making it such that the prior has little influence on the posterior distribution. We will calculate the posterior probability that *θ* exceeds 0.8 in a single arm by calculating the size of the tail of the posterior distribution that is above 0.8.
