# Supplementary material for: Completion of isoniazid–rifapentine (3HP) for tuberculosis prevention among people living with HIV: Interim analysis of a hybrid type 3 effectiveness–implementation randomized trial
Source: PLoS Med. 2021 Dec 16;18(12):e1003875. doi: 10.1371/journal.pmed.1003875 (PMC8726462; doi:10.1371/journal.pmed.1003875)
Supplement: S1 Trial Protocol — TB, tuberculosis. (DOCX) [file pmed.1003875.s005.docx]

**Options for Delivering Isoniazid-Rifapentine (3HP) for TB Prevention (3HP Options Implementation Trial)**

**NIH/NHLBI R01HL144406**

**OFFICIAL**

**ClinicalTrials.gov Identifier:**

NCT03934931

**Sponsored by:**

NIH/NHLBI

**Unique Protocol ID:**

R01HL144406

**Principal Investigator(s):**

Adithya Cattamanchi MD, MAS

David W. Dowdy, PhD

Fred C. Semitala MB.Ch.B, M.Med, MPH, ImS

**Draft or Version Number:**

*Version 1.3*

**Day Month Year**

July 19, 2021

**Statement of Compliance**

The study will be carried out in accordance with Good Clinical Practice (GCP) as required by the following:

- *U.S. Code of Federal Regulations applicable to clinical studies (45 CFR 46)*
- *ICH GCP E6*
- *Completion of Human Subjects Protection Training*
- *NIH Clinical Terms of Award*

*Refer to:*

[*http://www.hhs.gov/ohrp/humansubjects/guidance/45cfr46.htm#46*](http://www.hhs.gov/ohrp/humansubjects/guidance/45cfr46.htm#46)

[*http://www.fda.gov/cder/guidance/959fnl.pdf*](http://www.fda.gov/cder/guidance/959fnl.pdf)

[*http://grants.nih.gov/grants/guide/notice-files/NOT-OD-01-061.html*](http://grants.nih.gov/grants/guide/notice-files/NOT-OD-01-061.html)

[*http://cme.cancer.gov/c01/*](http://cme.cancer.gov/c01/)

**Signature Page**

The signature below constitutes the approval of this protocol and the attachments, and provides the necessary assurances that this trial will be conducted according to all stipulations of the protocol, including all statements regarding confidentiality, and according to local legal and regulatory requirements and applicable US federal regulations and ICH guidelines.

Uganda Study Site Principal Investigator: *

Signed: ____________________________________ Date: _____________________ Fred C. Semitala MB.Ch.B, M.Med, MPH, ImS

Overall Study Principal Investigators: *

Signed: ____________________________________ Date: _____________________

Adithya Cattamanchi, MD, MAS

Signed: ____________________________________ Date: _____________________

David W. Dowdy, PhD

**Document History**

| **Version No.** | **Version Date** | **Description of Change** |
| --- | --- | --- |
| 1.0 | 2019-11-01 | - Initial release. |
| 1.1 | 2020-08-25 | - Inclusion of option of taking medication by fixed dose combination pills. - Inclusion of transport reimbursement range due to changes in transport costs due to COVID-19 pandemic. - Removal of details for analysis of implementation and cost-effectiveness outcomes, which are being developed in separate protocols. |
| 1.2 | 2020-11-20 | - Inclusion of Uganda National guidelines for women who become pregnant while on 3HP. |
| 1.3 | 2021-07-19 | - Removal of sputum testing for active tuberculosis for all patients at 16 months follow-up. - Inclusion of verbal informed consent for qualitative interviews. |

Table of Contents

[Overview 5](#_Toc68157119)

[Key Roles 6](#_Toc68157120)

[I. Protocol Synopsis 9](#_Toc68157121)

[II. Background 12](#_Toc68157122)

[Background information 12](#_Toc68157123)

[III. Study Objectives 14](#_Toc68157124)

[IV. Methods/Design 14](#_Toc68157125)

[Overview of Study Design 14](#_Toc68157126)

[Target Setting and Study Population 15](#_Toc68157127)

[Study Agents 15](#_Toc68157128)

[Study Arms 15](#_Toc68157129)

[Eligibility Criteria 16](#_Toc68157130)

[1. **Inclusion Criteria:** 16](#_Toc68157131)

[2. **Exclusion Criteria:** 17](#_Toc68157132)

[Recruitment and eligibility screening 17](#_Toc68157133)

[Written Informed consent 18](#_Toc68157134)

[Randomization 19](#_Toc68157135)

[Study Procedures (See Table 2 and Figure 1) 19](#_Toc68157136)

[***Day 1 (day of enrollment)*** 19](#_Toc68157137)

[***Weekly or Monthly during 3HP treatment*** 20](#_Toc68157138)

[***Post-3HP treatment*** 22](#_Toc68157139)

[Outcome Measures 23](#_Toc68157140)

[**Primary Outcome (Effectiveness of delivery options)** 23](#_Toc68157141)

[**Secondary Outcomes (Quantitative)** 23](#_Toc68157142)

[Statistical Considerations 25](#_Toc68157143)

[V. Data Management 27](#_Toc68157144)

[VI. Ethical Considerations 27](#_Toc68157145)

[VII. Abbreviations 31](#_Toc68157146)

[VIII. References 32](#_Toc68157147)

# **Overview**

| **Title** | Options for Delivering Isoniazid-Rifapentine (3HP) for TB Prevention (3HP Options Implementation Trial) |
| --- | --- |
| **Target Population** | Adults living with HIV infection (n=1656) |
| **Site(s)** | Mulago Immune Suppression Syndrome (AIDS) Clinic, Kampala, Uganda |
| **Study Design** | Three-arm open label parallel randomized implementation trial |
| **Study Duration** | 48 months |
| **Objectives** | **Primary Objective: To compare the uptake of 3HP under three delivery strategies:**   - Directly Observed Therapy (DOT); - Self-administered Therapy (SAT); or - Patient choice (with the help of a decision aid) of either DOT or SAT.   **Secondary Objectives:**   1. To estimate the costs and compare the cost-effectiveness of three strategies for delivering 3HP. 2. To identify processes and contextual factors that influence patient acceptance and completion of 3HP under each delivery strategy. 3. To identify clinic-level barriers to adoption and implementation of 3HP under each delivery strategy. 4. To determine the proportion of patients for whom 3HP treatment is discontinued due to adverse events/intolerance. 5. To determine the cumulative 16-month incidence of active TB in each arm. |

# **Key Roles**

**Principal Investigators**

Adithya Cattamanchi, MD, MAS

Associate Professor of Medicine, University of California San Francisco

San Francisco General Hospital

1001 Potrero Ave, Room 5K1

San Francisco, CA 94110

Phone: +1-415-206-5489

Fax: +1-415-695-1551

[adithya.cattamanchi@ucsf.edu](mailto:adithya.cattamanchi@ucsf.edu)

David W. Dowdy, PhD

Associate Professor, Johns Hopkins Bloomberg School of Public Health

615 N. Wolfe Street, Room E6531
Baltimore, MD 21205

[ddowdy1@jhmi.edu](mailto:ddowdy1@jhmi.edu)

**Uganda Principal Investigator**

Fred C. Semitala, M.B.Ch.B, M.Med, M.P.H., ImS

Makerere University, School of Medicine

Department of Internal Medicine

Kampala, Uganda

Phone: +256-755-553-004

[semitala@gmail.com](mailto:semitala@gmail.com)

**Co- Investigators**

Achilles Katamba, MBChB, MSc, PhD

Makerere University, School of Medicine

Clinical Epidemiology Unit, Department of Internal Medicine

Kampala, Uganda

Phone: +256-753-040-922

Fax: +256-(0)414-540-524

[axk95@case.edu](mailto:axk95@case.edu)

Anne Katahoire PhD

Makerere University, Child Health and Development Centre

Kampala, Uganda

[annekatahoire@yahoo.co.uk](mailto:annekatahoire@yahoo.co.uk)

Moses Kamya, M.B.Ch.B., M.Med, MPH, PhD

Makerere University, School of Medicine

Kampala, Uganda

Phone: +256-414-541-188

[mkamya@infocom.co.ug](mailto:mkamya@infocom.co.ug)

Noah Kiwanuka MBChB, MPH, PhD

Makerere University College of Health Sciences

Kampala, Uganda

Phone: +256-782-788036/+256-701-788036

nkiwanuka@musph.ac.ug

Patrick Phillips PhD, MS, MA

Assistant Professor, University of California San Francisco

San Francisco General Hospital

1001 Potrero Ave, Room 5K1

San Francisco, CA 94110

Phone: +1-415-206-3960

[Patrick.Phillips@ucsf.edu](mailto:Patrick.Phillips@ucsf.edu)

Payam Nahid MPH, MD

Professor of Medicine, University of California San Francisco

San Francisco General Hospital

1001 Potrero Ave, Room 5K1

San Francisco, CA 94110

Phone: +1-415-206-5464

[pnahid@ucsf.edu](mailto:pnahid@ucsf.edu)

**Table 1. Timeline of Activities**

| **Timeline** | **2019** | | | | | **2020** | | | | **2021** | | | | **2022** | | | |
| --- | --- | --- | --- | --- | --- | --- | --- | --- | --- | --- | --- | --- | --- | --- | --- | --- | --- |
|  | **Q1** | **Q2** | **Q3** | **Q4** | **Q1** | | **Q2** | **Q3** | **Q4** | **Q1** | **Q2** | **Q3** | **Q4** | **Q1** | **Q2** | **Q3** | **Q4** |
| RCT of 3HP delivery strategies (enrollment and follow-up) |  |  |  |  | ● | | ● | ● | ● | ● | ● | ● | ● |  |  |  |  |
| Quantitative analysis of process metrics and patient surveys |  |  |  |  | ● | | ● | ● | ● | ● | ● | ● | ● | ● | ● |  |  |
| Qualitative data collection and analysis |  |  |  |  | ● | | ● | ● | ● | ● | ● | ● | ● | ● | ● |  |  |
| Cost data collection and costing analyses |  |  |  |  | ● | | ● | ● | ● | ● | ● | ● | ● |  |  |  |  |
| Cost-effectiveness modeling |  |  |  |  |  | |  |  |  |  |  | ● | ● | ● | ● | ● | ● |

1. **Protocol Synopsis**

The Options for Delivering Isoniazid-Rifapentine (3HP) for TB Prevention (3HP Options Implementation Trial) study will be a three-arm, open-label, parallel, randomized trial. This hybrid effectiveness-implementation trial will be conducted among people living with HIV infection (**PLHIV**) enrolled in HIV/AIDS care at the Mulago Immune Suppression Syndrome (*i.e.,* HIV/AIDS) clinic in Kampala, Uganda. The overall objective of this study is to identify a patient-centered delivery strategy that will facilitate acceptance and completion of a three-month (12-dose) regimen of weekly rifapentine (**RPT**) and isoniazid (**INH**) by PLHIV enrolled in routine HIV/AIDS care in a high HIV/TB burden country. The primary outcome will be acceptance and completion of 3HP. Additional objectives will be to evaluate the implementation and cost-effectiveness of each delivery strategy.

**Primary Objective:**

To compare the uptake of 3HP under three delivery strategies: 1) Facilitated DOT; 2) Facilitated SAT; and 3) Informed patient choice (using a decision aid) between facilitated DOT and facilitated SAT. The primary outcome will be defined as the proportion of eligible participants who accept treatment and take at least 11 of 12 doses of RPT/INH within 16 weeks of study enrollment.

**Secondary Objectives:**

1. To estimate the costs and compare the cost-effectiveness of three strategies for delivering 3HP.
2. To identify processes and contextual factors that influence patient acceptance and completion of 3HP under each delivery strategy.
3. To identify clinic-level barriers to adoption and implementation of 3HP under each delivery strategy.
4. To determine the proportion of patients for whom 3HP treatment is discontinued due to adverse events/intolerance.
5. To determine the cumulative 16-month incidence of active TB in each arm.

**Study Design:**

Participants will be randomized to one of three arms to receive latent tuberculosis infection (**LTBI**) treatment with once weekly isoniazid (INH) and rifapentine (RPT) for 12 weeks given by either facilitated DOT, facilitated SAT, or an informed choice between DOT and SAT (with the assistance of a decision aid tool).

**Study Agents:**

Participants in all arms will receive the following medications approved for TB preventive therapy by the World Health Organization (**WHO**):

- RPT 900 mg and INH 900 mg once-weekly x 12 doses
- Pyridoxine (vitamin B_6_) 50 mg offered once weekly, as recommended for 3HP, to protect against isoniazid-related peripheral neuropathy

**Study Population/Sample Size:**

We will enroll 1656 PLHIV (552/arm) aged 18 years and above accessing HIV/AIDS care at the Mulago Immune Suppression Syndrome Clinic who meet all study inclusion criteria and none of the exclusion criteria.

**Outcomes:**

- - - 1. **Primary Outcome:**
- The proportion of eligible participants who accept treatment and take at least 11 of 12 once weekly doses of RPT/INH within 16 weeks of study enrollment.

1. **Secondary Outcomes categorized using the RE-AIM framework:**

| **RE-AIM Domain** | **Outcome** |
| --- | --- |
| **Reach** | - Proportion of eligible PLHIV offered 3HP who accept to initiate treatment (by age, gender, CD4 stratum, viral load suppression). |
| **Effectiveness/**  **Cost-effectiveness** | - Incremental cost of each delivery strategy per disability adjusted life year (**DALY**) averted. - The incremental health system cost per DALY averted. - The incremental patient cost per DALY averted. - Proportion of participants who initiate 3HP for whom treatment is discontinued due to adverse events or intolerance. - Cumulative 16-month incidence of active TB. |
| **Adoption** | - Proportion reimbursed overall and on the same day as each 3HP clinic visit. - Time spent at each clinic visit. - Proportion of SMS or IVR phone call reminders delivered to participants for clinic visits or medication dosing - Proportion of SMS or IVR phone call reminders delivered to participants for missed appointments - Proportion of participants screened for active TB during DOT or refill visits - Proportion of participants screened for side effects during DOT or refill visits.   **Participants taking 3HP by SAT only**   - Proportion of doses confirmed using digital adherence technology. Doses directly observed (*i.e.*, during initial or refill visits) will not be included in the denominator. - Proportion of SMS or IVR phone call reminders delivered to participants following missed doses - Proportion of weekly SMS or IVR phone call check-ins delivered to participants - Proportion of responses to weekly SMS or IVR phone call check-ins received from participants - Proportion of participants who receive appropriate follow-up (phone call or home visit) for lack of response/negative response to weekly check-in SMS or IVR phone call |
| **Implementation** | - Total direct and indirect patient costs related to TB preventive care services. - Self-reported patient barriers to TB preventative care services. - Patient satisfaction with TB preventive care services. - Provider- and clinic-level barriers to delivery of 3HP. |
| **Maintenance** | Not applicable. |

**Assessment of Primary Outcome**:

The primary outcome of 3HP acceptance and completion will be assessed 16 weeks after study enrollment. According to our study definition, eligible participants who initiate and take a minimum of 11 of the 12 total 3HP doses within the 16-week time period will have met criteria for treatment acceptance and completion. Study staff will assess medication dosing using clinic records for participants taking 3HP by DOT and using a combination of 99DOTS (Everwell Health Solutions, India) digital medication adherence technology records and pill counts at refill visits for participants taking 3HP by SAT.

# **Background**

## **Background information**

Tuberculosis (**TB**) kills >350,000 people living with HIV (**PLHIV**) each year, which accounts for 25% of all HIV-associated deaths. Preventive therapy can reduce TB incidence by 30-50% and the risk of death or severe illness by 35%.^1^ Models have suggested that scaling up preventive therapy is the only way that aggressive targets for TB control can be achieved.^2^ The World Health Organization (**WHO**) and the US President’s Emergency Plan for AIDS Relief (**PEPFAR**) unequivocally support providing TB preventive therapy to all PLHIV, and the WHO lists TB preventive therapy as a core service of National AIDS Programs.

**Treatment of Latent Tuberculosis Infection (LTBI)**

Implementation of treatment of latent tuberculosis infection (**LTBI**), or TB preventive therapy, for PLHIV has been extremely disappointing. In 2015, fewer than 1 million out of the nearly 37 million PLHIV worldwide received TB preventive therapy. Historically, challenges to scale-up have included lack of clear guidelines for ruling-out active TB or monitoring adherence, a requirement to document infection prior to treatment, and a perceived risk of fueling drug resistance.^3^ These challenges are actively being addressed at the policy level and through increased political commitment to TB prevention. Major patient-level barriers to scale-up of TB preventive therapy remain, however, including concerns about toxicity and the burden of taking daily pills over a prolonged duration. Completion rates have generally failed to reach even 50% in the context of national scale-up programs^4,5^, and in Uganda the proportion lost before completing treatment increases steadily with each additional month of therapy.^6^

**Shorter LTBI Treatment Regimens**

Preventive therapy for tuberculosis has traditionally consisted of 6-9 months of daily isoniazid (**INH**).^7^ Although known to reduce TB incidence and mortality among PLHIV, uptake of this protracted regimen in high-burden countries remains very poor.^8^ A three-month (12-dose) regimen of weekly isoniazid and rifapentine (**3HP**) has been approved based on randomized trials showing equivalent efficacy and better tolerability compared to 9 months of daily isoniazid.^9,10^ Pharmacodynamic studies have shown that rifapentine can be co-administered with efavirenz – the backbone of most first-line antiretroviral therapy (**ART**) regimens in Africa – and with integrase inhibitors such as raltegravir without clinically meaningful changes in mid-dosing concentrations and/or HIV viral load.^11-13^ Dolutegravir (DTG) can be given with 3HP without dose adjustment, according to data from the DOLPHIN trial^14^. No safety concerns emerged from a trial of daily rifapentine for TB prevention among PLHIV on efavirenz- or nevirapine-based ART regimens^15^. Thus, 3HP is compatible with multiple ART regimens already widely in use or likely to be available at scale in most high-burden countries. Based on these data, WHO guidelines now recommend 3HP for PLHIV and several high burden countries have already made high-level policy decisions to scale up 3HP among PLHIV nationwide.^16^

**Self-administered Therapy (SAT) vs. Directly Observed Therapy (DOT) for LTBI**

The best approach to delivering 3HP remains uncertain. Directly observed therapy (**DOT**) has resulted in higher adherence in some settings^17^ and provides a regular opportunity to monitor adverse reactions and address patient concerns, but clinic visits can be prohibitively costly and time-consuming for patients, and high levels of acceptance and completion may not be achievable in the context of routine HIV/AIDS care. Self-administered therapy (**SAT**) overcomes some of these barriers but may lead to lower adherence in some contexts^17^ and delayed recognition of adverse events. Furthermore, patients may have different preferences for DOT versus SAT.

With either DOT or SAT, a major advantage of a 12-dose regimen is that it becomes feasible to *facilitate* adherence to each dose. Recently, completion of 3HP when given by DOT (with financial incentives) or SAT (with or without weekly SMS reminders) was compared at clinical trial sites in the U.S., Spain, Hong Kong and South Africa.^17^ The non-inferiority of SAT (74-76% completion) relative to DOT (87% completion) could not be demonstrated, and SAT performed worst in South Africa (<50% completion). Now, trials using theory-informed approaches to treatment facilitation with DOT and SAT are urgently needed. To date, no previous study has: a) evaluated whether high levels of 3HP treatment completion can be achieved – by DOT or SAT – in the context of routine HIV/AIDS care in sub-Saharan Africa; b) adopted a theory-informed approach to optimizing 3HP delivery through treatment facilitation; or c) clearly identified the most effective and cost-effective 3HP delivery strategy for high-burden settings. In order to realize the promising potential of 3HP to reduce TB burden among PLHIV, there is an *urgent need* to optimize 3HP delivery at HIV/AIDS clinics in sub-Saharan Africa, where TB remains the leading cause of death.^8^

**Monitoring Adherence to LTBI Medication**

Innovative approaches to promoting and monitoring adherence to TB medications are now available. In particular, digital adherence technologies have been proven to be effective and well accepted by both patients and providers in low-resource settings^18^. 99DOTS (Everwell Health Solutions, India) is a novel, simple and low-cost ($4-6/patient at scale) technology whereby medications are packaged alongside hidden toll-free phone numbers, enabling patients to make toll-free calls to confirm medication dosing. Clinic staff can access adherence data for individual patients through a web dashboard and mobile phone application. Short message service (**SMS**) reminders (to patients for dosing and to providers to follow-up on patients who have missed doses), interactive voice recorded phone calls, and interactive two-way messaging are also core features of the platform. 99DOTS thus enables real-time identification of patients who miss doses for further follow-up through an open-source platform, has been used to monitor adherence of TB patients in similar resource-constrained settings.

**Shared Decision Making in the Context of LTBI**

Shared decision-making (**SDM**) is a promising strategy to improve 3HP delivery to PLHIV in sub-Saharan Africa. SDM is an important aspect of patient-centered care, which the Institute of Medicine has identified as one of six key components of high-quality health care.^19^ Key characteristics of shared decision-making include: a) information sharing; b) participation by all parties in consensus building about preferred treatment; and c) reaching an agreement about treatment.^20^ SDM interventions such as decision aids have been shown to improve knowledge and accuracy of risk perceptions, decisional conflict, match between personal values and choice, and compliance with medical care.^21,22^ They are most useful for decisions in which there is more than one medically reasonable option (*e.g.,* receiving 3HP by DOT vs. SAT), such that the choice between options aligns with the patient’s values and preferences.^23^ SDM interventions have been shown to be feasible and more beneficial to disadvantaged groups than to those with higher literacy, education and socioeconomic status, particularly when tailored to the situation^24^ – suggesting that SDM could be very effective in resource-limited settings. SDM is also consistent with the longstanding tradition of treatment literacy and patient empowerment in HIV programs, but requires rigorous testing before wide implementation.^25^

1. **Study Objectives**

Ultimately, interventions must work for patients to be effective at the population level. The overall objective of this study is to identify a patient-centered strategy that will facilitate 3HP uptake by PLHIV in the context of routine HIV/AIDS care in a high HIV/TB burden country. Our central hypothesis is that offering PLHIV an informed choice between DOT and SAT delivery strategies that are optimized to overcome key barriers to treatment adherence will result in greater acceptance and completion of 3HP.

**Primary Objective:**

To compare the uptake of 3HP under three delivery strategies: 1) Facilitated DOT; 2) Facilitated SAT; and 3) Informed patient choice (using a decision aid) between facilitated DOT and facilitated SAT. The primary outcome will be defined as the proportion of eligible participants who accept treatment and take at least 11 of 12 doses of RPT/INH within 16 weeks of study enrollment.

**Secondary Objectives:**

1. To estimate the costs and compare the cost-effectiveness of the three strategies for delivering 3HP.
2. To identify processes and contextual factors that influence patient acceptance and completion of 3HP under each delivery strategy.
3. To identify clinic-level barriers to adoption and implementation of 3HP under each delivery strategy.
4. To determine the proportion of patients for whom 3HP treatment is discontinued due to adverse events/intolerance.
5. To determine the cumulative 16-month incidence of active TB in each arm, categorized as *definite* (positive sputum Xpert MRB/RIF or culture) or *probable* (TB medications started at the discretion of a clinician, with evidence of subsequent improvement).

# **Methods/Design**

## **Overview of Study Design**

We will conduct a pragmatic randomized trial of three optimized strategies for delivering 3HP. Eligible participants will be randomized to one of three arms to receive LTBI treatment with once weekly INH and RPT for 12 weeks given by either facilitated DOT, facilitated SAT, or an informed choice between facilitated DOT and facilitated SAT (with the assistance of a decision aid tool). Research staff will be responsible for participant consent, randomization, questionnaire and survey administration, pre-treatment counseling and post-treatment follow-up. To facilitate evaluation of 3HP delivery under routine clinic conditions, research staff will not interact with participants between treatment initiation and either discontinuation or completion. Thus, Mulago Immune Suppression Syndrome (**ISS**) clinic (*i.e.,* Mulago AIDS clinic) staff will perform all activities related to 3HP treatment during DOT or refill visits, including screen participants for side effects, screening participants for active TB, dispensing medicines and, for participants taking 3HP by SAT, reviewing electronic dosing records and responses to weekly SMS or IVR phone call check-ins. Embedded mixed methods and health economic analyses will assess the implementation and cost/cost-effectiveness of the three delivery strategies (Figure 1).


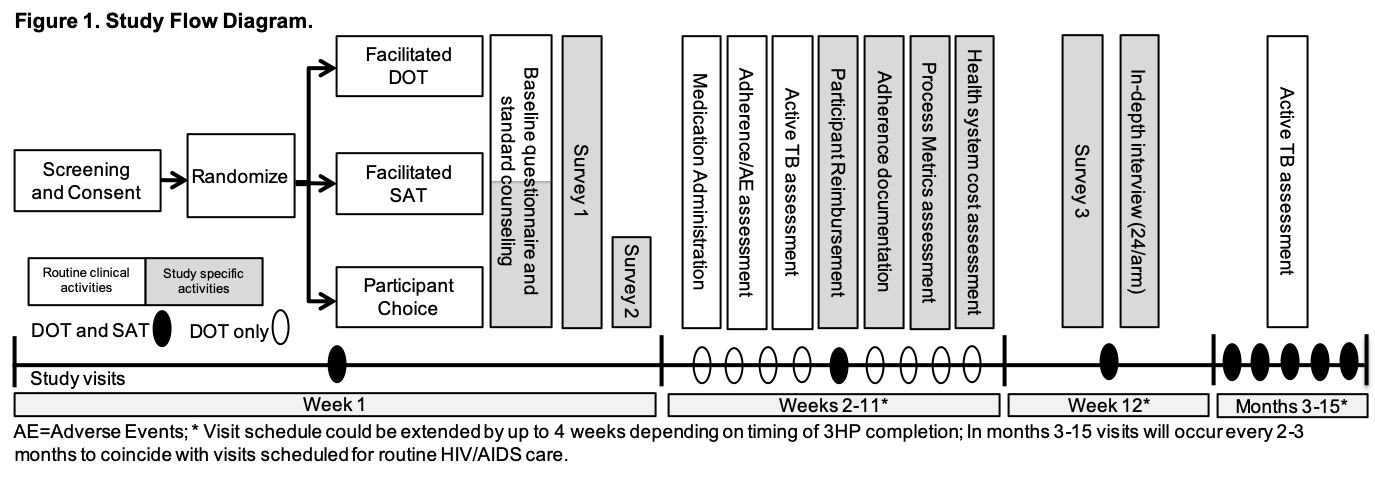


## **Target Setting and Study Population**

The target setting is the Mulago ISS clinic, a large urban referral clinic (16,000 clients enrolled and 300 new clients registered monthly) that accepts patients from multiple HIV testing sites in Kampala, Uganda, and is run by the PEPFAR-funded Makerere University Joint AIDS Program (**MJAP**). The target population for this trial is adult PLHIV enrolled in HIV/AIDS care. The unit of randomization and analysis will be 1656 PLHIV receiving HIV/AIDS care at the Mulago ISS clinic. 3HP is a recommended regimen in Uganda and the Mulago ISS clinic will procure the medicines from the Global Drug Facility or local drug distributors where available.

## **Study Agents**

Irrespective of study arm, all participants will receive the following medications approved for TB preventive therapy by the WHO: RPT 900 mg and INH 900 mg. Depending on availability, the medications will be provided either as individual RPT (6 pills, each 150 mg/tablet) and INH (3 pills, each 300 mg/tablet) tablets or as fixed-dose combination tablets (3 pills, each containing 300 mg RPT and 300 mg INH) to take once-weekly for 12 weeks. We will not adjust doses of RPT or INH based on weight for this study. Supplies of RPT will be procured from the Global Drug Facility. RPT tablets will be provided in commercially marketed blister packs which contain 8 RPT tablets per pack. INH is registered with the Uganda National Drug Authority (**NDA**) and will be sourced locally from drug distributors in Uganda. Supplies of the fixed dose combination INH-RPT tablets, which have been approved for use by the Uganda NDA, will be procured from the Global Drug Facility or directly from the sole manufacturer (Macleods Pharmaceuticals, India).

The total incidence of all adverse effects from isoniazid is approximately 5%. Nervous system reactions, usually peripheral neuritis, are the most common side effect. The risk of peripheral neuritis is dose dependent, and the risk increases for persons who are malnourished or predisposed to neuritis by other illnesses. For these reasons, we will offer participants concomitant administration of pyridoxine (vitamin B_6_) 50 mg offered weekly, as recommended for 3HP, to protect against isoniazid-related peripheral neuropathy. Pyridoxine is registered with the Ugandan NDA and will be sourced locally from drug distributors in Uganda.

## **Study Arms**

**Arm 1: Facilitated DOT**

Participants randomized to the facilitated DOT arm will attend the Mulago ISS clinic on a weekly basis to ingest 3HP medication under direct observation. DOT will be defined as a designated clinic staff member observing ingestion of each dose of 3HP. Additionally, participants randomized to facilitated DOT will receive:

DOT cards with instructions to present directly to the pharmacy for a pharmacy-only visit, without the need to wait in the general queue.

Automated SMS or interactive voice response (**IVR**) phone call reminders at no cost to participants the day before each appointment.

A fixed level of reimbursement (~$5-8/visit) for each weekly visit, conditional on either directly observed therapy or evidence of an adverse event that would preclude further treatment.

Participants who fail to return for a scheduled appointment within 24 hours will receive an SMS or IVR phone call reminder for three consecutive days after the missed appointment.

**Arm 2: Facilitated SAT**

Participants randomized to the facilitated SAT arm will take their first dose of medication under direct observation and be provided with a 4-week supply of 3HP to take weekly via self-administration. Participants will be asked to return to the Mulago ISS clinic after completing their 5^th^ dose to review adherence data with the Mulago ISS clinic pharmacy technician and receive 5 additional doses of 3HP (doses 7-11). At the scheduled refill visit (dose 6) and the end-of-treatment visit (dose 12), participants will ingest 3HP doses under direct observation. Additionally, participants will receive:

1. 99DOTS-based digital adherence technology to monitor and promote adherence.
2. Weekly check-ins via two-way SMS or IVR phone call for dosing reminders and messaging asking “Are you well?” (or other similar message, as determined in our formative research phase), to serve as an alternative to weekly visits with a health worker to inquire about potential side effects
3. A fixed level of reimbursement (~$5-8/visit) for the refill and end-of-treatment visit, conditional on either directly observed therapy or evidence of an adverse event that would preclude further treatment.

Participants who fail to return for a scheduled appointment within 24 hours will receive an SMS or IVR phone call reminder for three consecutive days after the missed appointment.

**Arm 3: Patient Choice between facilitated DOT and facilitated SAT**

Participants randomized to the Patient Choice between facilitated DOT and facilitated SAT arm will be offered a choice between arms 1 and 2. A research nurse will review each section of the decision aid with participants, discuss values and preferences, and, after addressing any questions, ask participants to select facilitated DOT or facilitated SAT. Participants will have the option to switch between DOT and SAT at any time. The reason for switching and time spent under each strategy will be recorded.

## **Eligibility Criteria**

To be included in the randomized trial, patients will have to meet all of the following inclusion criteria and none of the exclusion criteria:

1. **Inclusion Criteria:**
2. HIV-positive client engaged in care at the Mulago ISS clinic
3. Weight ≥40kg
4. Age 18 years or older
5. Capacity to provide informed consent in English or Luganda

(From our experience conducting similar studies at the Mulago ISS clinic, the majority of patients receiving care at the clinic are able to communicate using the Luganda language. For example, while conducting patient Knowledge, Attitudes and Practices (KAP) interviews during the formative phase, 21/25 (84%) participants opted for Luganda and only four of the 25 opted for English as the language for the interview. Therefore, in addition to Luganda, we selected English as the other preferred language for the study)

1. **Exclusion Criteria:**
   1. Suspicion of active TB based on positive WHO symptom screen AND elevated POC CRP, or current or planned TB treatment
   2. Actively taking an antiretroviral medication contraindicated for use with rifapentine under contemporary WHO or Ugandan policy
   3. Contact of a TB patient with known resistance to isoniazid or rifamycins
   4. Women who are pregnant, breast feeding or intending to get pregnant in the next 120 days. Note: women who become pregnant while on 3HP will be recommended to continue and complete 3HP treatment per Uganda 2020 Uganda National Guidelines, and will be monitored for side effects as per the study protocol ^26^
   5. Prisoners
   6. Previously completed treatment for active TB or at least 6 months of isoniazid preventive therapy within past 2 years
   7. Not intending to remain within 25 km of the Mulago ISS clinic during the study period or to receive further care at the Mulago ISS clinic
   8. Lack of access to a mobile telephone or lack of willingness to receive SMS reminders/IVR phone calls
   9. Pre-existing documentation of clinical liver disease.
   10. History of sensitivity or intolerance to isoniazid or rifamycins
   11. Another household member already enrolled in the study (household members cannot be effectively randomized to different arms)
   12. Actively taking medication contraindicated for use with rifamycin (*e.g.,* warfarin, phenytoin)

Mixed methods and health economic sub-studies will include a subset of participants enrolled in the trial, as well as clinic administrators and clinicians (clinical officer, doctor, nurse, pharmacist, peer educators and counselors) involved in 3HP delivery at the Mulago ISS clinic.

## **Recruitment and eligibility screening**

PLHIV will be recruited through Mulago ISS clinic providers, who will refer interested clients to study staff for eligibility screening, or directly from the patient waiting area by a trained research nurse. In order to be considered for study entry, all interested clients must have completed the following screening evaluations/documentation:

A baseline medical history and clinical evaluation, including:

Patient demographics (*i.e.,* sex, age)

Access to mobile telephone/willingness to receive SMS reminders/IVR phone calls

Intention to remain within 25 km of the Mulago ISS clinic during the study period or to receive further care at the Mulago ISS clinic

Height and weight

Previous treatment for LTBI (*i.e.,* if patient has previously completed a full course of 3HP or at least 6 months of isoniazid preventive therapy)

Evaluation of contraindications for 3HP treatment, including:

Active treatment with an antiretroviral medication contraindicated for use with rifapentine under contemporary Ugandan policy

1. Current breastfeeding, pregnancy, or a plan to get pregnant within 120 days of enrollment (a pregnancy test in women of childbearing potential must be obtained <14 days prior to enrollment). Note: women who become pregnant while on 3HP will be recommended to continue and complete 3HP treatment per Uganda 2020 National Guidelines, and will be monitored for side effects as per the study protocol.
2. Contact of a case with known resistance to INH or rifapentine.
3. Risk for underlying liver disease which includes pre-existing documentation of clinical liver disease.

Laboratory tests, including:

A blood or urine pregnancy test done on all women of reproductive potential.

Evaluation for active TB

Symptom screen and if positive POC CRP measurement via finger prick blood sample

Clients who do not meet the eligibility criteria at the time of screening may be eligible at a later time. Therefore, clients can be subsequently re-screened (i.e., screened more than once) in order to be eligible for the study.

## **Written Informed consent**

A research nurse will read aloud from an institutional review board (**IRB**)-approved consent form in the client’s preferred language (English or Luganda). A copy of the consent form in the client’s preferred language will also be given to the client. If the client is illiterate, a third party of the client’s choice will be asked to witness the consent. While participants will be encouraged to ask additional questions throughout the consent process, at a minimum the following areas will be addressed.

- Participant rights
- Purpose
- Procedures
- Risks and side effects
- Benefits
- Alternatives
- Questions

At the conclusion of discussions of these topics, the eligible participant will be asked, “Do you wish to participate?” after making it clear that a decision to consent or not will not affect the client or any of his or her family members’ right to receive standard prevention, care, and treatment for TB or HIV. If the client agrees, he or she will be asked to sign his or her name on the consent form, or, if illiterate, to provide witnessed consent by someone who is not part of the research team, as described above. In all cases, the nurse will also sign his/her name as a witness. The consent forms, which will be approved by IRBs in the U.S. and Uganda, will be translated from English into Luganda, and back-translated into English as required by IRBs to be sure that no significant language or concepts are lost in translation. If the client does not wish to participate, the nurse will thank the patient. Clients who are unable to communicate (because of altered mental status or inability to speak English or Luganda) may be excluded because of a lack of ability to provide informed consent.

**Verbal Informed consent to participate in qualitative telephone interviews**

Up to 72 participants (24/arm) will be purposively selected to participate in in-depth interviews to identify processes and contextual factors that influenced their acceptance and completion of 3HP under each delivery strategy. If necessary, some in-depth interviews will be conducted via telephone calls made by study staff to the selected participants. Verbal informed consent to participate in audio recorded telephone interviews will be obtained from the selected participants interviewed by phone.

## **Randomization**

All interested participants who meet eligibility criteria and consent to be included in the study will be randomized via a computer-generated algorithm to one of three arms: facilitated DOT, facilitated SAT, and patient choice between facilitated DOT and facilitated SAT (with the help of a decision aid tool). Randomization will be performed using computer-generated random permuted blocks of variable size between 9, 12 and 15, with an equal allocation ratio between the three arms. Randomization numbers will be generated using a statistical software package and individual random assignment sheets will be placed and sealed in opaque envelopes in batches of multiple blocks to minimize predictability that would occur if single blocks were used. Generation of randomization numbers and their concealment will be done by a US-based Study Biostatistician who will not have contact with participants. The numbers will be passed on to the local Study Biostatistician for custody and onward transfer to Study Coordinator in numbers corresponding to the expected enrolments for given time periods. Accountability for released randomization number containing envelopes will be given prior to release of subsequent ones. Each eligible participant will be asked by a Study Coordinator or his/her designee to select an envelope which s/he will open to see the randomization number and study arm. This will help to avoid/mitigate participants from accusing the research team of selecting for them a study arm. All randomization numbers will be monitored daily by the Study Coordinator and weekly by the local Biostatistician and fortnightly by the US-based via a database at a data management center. Recruitment will be continued until 1656 participants are randomized and offered treatment (expected to be completed within 2 years).

## **Study Procedures (See Table 2 and Figure 1)**

### ***Day 1 (day of enrollment)***

1. **Baseline questionnaire**: A research nurse will administer a baseline questionnaire to collect demographic and clinical information. The research nurse will also register all participants into the 99DOTS software to receive automated SMS/IVR phone call reminders (all participants) and to record dosing history (Facilitated SAT participants only). Participants will be asked to provide up to three telephone numbers for contact. The research nurse completing patient registration will call each telephone number provided by the patient to confirm number accuracy. The study staff member will inform the participant that these numbers will be used to send toll-free, automated SMS reminders for scheduled clinic visits the day before each appointment (all participants) and for medication dosing on the scheduled dosing day (Facilitated SAT participants only).
2. **Pre-treatment counseling:** A research nurse will provide standardized counseling to participants in all arms using a handout to provide information about TB and LTBI and the risks and benefits of LTBI treatment with 3HP. All participants will be counseled about the signs and symptoms of study drug-related toxicity, and will be instructed to contact a study staff member or clinic provider immediately if any signs or symptoms occur (all participants) and to discontinue the study medications (Facilitated SAT participants). The counseling will include information about the increased risk of liver toxicity with alcohol use and the importance of abstaining from alcohol use during 3HP treatment. For clients randomized to the choice arm, the research nurse will also use a standardized decision aid to review the two options for 3HP delivery.

After addressing any questions, the research nurse will ask if the client is willing to initiate 3HP (and also the mode of delivery if the client is in the choice arm). The research nurse will then review the details of the 3HP delivery strategy to which the client has been randomly assigned or chosen (if randomized to the choice arm). For SAT, this will include information on how to store 3HP medications, how to properly take 3HP medication each week, how to make phone calls each week to confirm dosing, how to respond to IVR phone call/SMS messages, how to recognize and report side effects and potential adverse events, and when to return for the refill visit. For DOT, this will include information on when and where to come for DOT visits each week, IVR phone call reminders for weekly clinic visits and how to recognize and report side effects and potential adverse events. Of note, participants who decline to initiate 3HP after randomization will be counted as not accepting/completing treatment in the primary (intent-to-treat) analysis and will be followed over time for clinical outcomes.

1. **Survey 1**: A research nurse will administer a survey to all participants to assess a) uptake of key messages delivered as part of standardized counseling about TB preventive therapy and b) costs associated with accessing the clinic. Cost data collection will be based on the WHO handbook for TB patient costing surveys, which includes family and coping costs, and which we have adapted and used in Uganda.^27^
2. **Survey 2**: Participants randomized to the choice arm will be administered a brief questionnaire to document reasons for their choice of delivery strategy.
3. **Medication administration:** Regardless of study arm, all participants will be referred to the Mulago ISS clinic pharmacy technician after completion of the above research activities to take their first dose of 3HP under direct observation. The pharmacy technician will record the time and date of medication ingestion in the clinic 3HP dosing register.
   1. **Facilitated DOT participants**: After ingesting their first dose, participants will be instructed to select a date and time to attend the Mulago ISS clinic on a weekly basis. Participants will then be given a DOT card with instructions to present directly to the pharmacy for a pharmacy-only visit, without the need to wait in the general queue on their next visit date, and every visit date thereafter. Participants will be informed that they will be reimbursed at a fixed rate for weekly visits.
   2. **Facilitated SAT participants**: After ingesting their first dose, participants will be asked to demonstrate knowledge of the appropriate dosing of 3HP, common side effects, and how to confirm dosing by making calls using the toll-free numbers. The patient will then be given sufficient pills for four doses of rifapentine and isoniazid. Participants will be asked to return to the Mulago ISS clinic at Week 6 with remaining pills (if any) Participants will be reminded that the scheduled Week 6 visit will be reimbursed at a fixed rate.

### ***Weekly or Monthly during 3HP treatment***

The following *routine TB preventive care activities* will be performed by Mulago ISS clinic staff during all 3HP DOT or refill visits:

1. **Adherence assessment:** The pharmacy technician will record in the clinic 3HP dosing register whether patients swallowed all prescribed pills including date and time of ingestion and, for patients taking 3HP by SAT, the technician will determine the number of doses taken based on 99DOTS medication adherence reports, pill counts and the participant’s self-report. In addition, the pharmacy technician will receive an automated SMS when a participant does not return for a scheduled dosing visit (all participants) or does not confirm dosing by calling the toll-free number within 24 hours of a scheduled 3HP dose (Facilitated SAT participants). The technician will be encouraged to call such participants to inquire about the reason for not taking their scheduled dose and provide additional counseling as needed. Participants will be considered to have failed to complete treatment within 16 weeks if they miss 6 or more doses during 3HP treatment.
2. **Adverse events screening**:
   1. **All participants**: A standardized checklist-based survey will be used to assess for symptoms of hepatotoxicity or other adverse events (*e.g.,* rash, peripheral neuropathy). The Mulago ISS clinic pharmacy technician will administer the survey at each scheduled clinic visit and at any unscheduled clinic visits if participants present due to concerns about toxicity. Participants with potential adverse events based on the survey will be referred to Mulago ISS clinic clinicians, who will make all decisions regarding further evaluation and treatment continuation or discontinuation. For those with potential symptoms of hepatotoxicity (e.g., abdominal pain, nausea, vomiting, unexplained fatigue or jaundice) this could include a blood draw to assess for elevated liver function tests. Note: women who become pregnant while on 3HP will be recommended to continue and complete 3HP treatment, and will be monitored for side effects as per routine procedures.
   2. **Facilitated SAT**: In lieu of weekly clinic visits, participants taking 3HP by Facilitated SAT will receive a weekly free (at no cost to them), automated SMS check-in message or IVR phone call asking “Are you well?” (or other similar message, as determined in our formative research phase) with previous instruction to respond “Yes” or “No”. All incoming SMS messages will be automatically reimbursed. The Mulago ISS clinic pharmacy technician will receive an automated SMS when a participant responds “No” or does not respond within 24 hours and will call the participant up to three times and ascertain whether he or she is having side effects using the standardized checklist-based survey. Participants who are unable to be contacted by phone will be visited at home by a community health worker (**CHW**). Participants with potential adverse events based on the survey will be referred to Mulago ISS clinic clinicians, who will make all decisions regarding further evaluation and treatment continuation or discontinuation. For those with potential symptoms of hepatotoxicity (e.g., abdominal pain, nausea, vomiting, unexplained fatigue or jaundice) this could include a blood draw to assess for elevated liver function tests.
3. **Active TB assessment:** The pharmacy technician will perform symptom screening using the Mulago ISS clinic intensified case finding form, and refer symptomatic patients to Mulago ISS clinicians for further evaluation. The clinician will make all decisions regarding TB testing, initiation of TB treatment, and continuation/discontinuation of 3HP.
4. **Medication administration:**
   1. **Facilitated DOT:** For all scheduled visits, medications will be prepared in advance. The pharmacy technician will address any patient questions about treatment, confirm continued interest in taking medications, provide INH and RPT tablets (as loose pills or fixed dose combination pills), with or without pyridoxine, and observe medication ingestion.
   2. **Facilitated SAT participants:** At weeks 6 and 12, participants will return to the Mulago ISS clinic. The pharmacy technician will address any patient questions about treatment, confirm continued interest in taking medications, provide INH and RPT tablets (as loose pills or fixed dose combination pills), with or without pyridoxine, and observe medication ingestion. At the week 6 visit, the technician will also provide additional doses of 3HP until the end-of-treatment visit and remind the patient about side effects.
5. **Participant reimbursement**: At each visit, the pharmacy technician will give DOT participants cash reimbursement for expenses related to transportation and food. SAT participants will be reimbursed at the same fixed level as DOT participants but only at their refill visit at week 6 and end-of-treatment visit at Week 12. A fixed level of reimbursement will be determined in our formative assessment and based off of current local transportation costs [which have doubled during the COVID pandemic] but will total approximately US$5-8 (15,000-30,000 USh) per visit. Receipt of this reimbursement will be conditional on either directly observed therapy or evidence of an adverse event that would preclude further treatment.

The following *study-specific activities* will be performed by research staff during 3HP DOT or refill visits:

1. **Adherence documentation**: Research staff will extract adherence data from clinic records and the 99DOTS server (SAT only) using a standardized form on a weekly basis. For participants in the SAT arm, self-report of adherence and pill counts will also be performed at the end-of-treatment visit. Self-report will primarily be used to understand reasons behind missed doses, and will only be used to measure adherence in the absence of any data from the 99DOTS server and pill counts.

In addition, time-and-motion studies and cost surveys will be conducted with selected participants in each arm along with detailed budgetary analysis to enable costing analyses. Process metrics will also assessed to determine whether intervention components were delivered as intended.

### ***Post-3HP treatment***

All participants will be aggressively traced and asked to return to clinic (reimbursed in the usual fashion) after completion of their final medication dose, or at 16 weeks post-enrollment (for participants not completing treatment).

The following *routine TB preventive care activities* will be performed by Mulago ISS clinic staff during a routine HIV/AIDS care clinic visit:

1. **Follow-up assessment:** Participants will be screened for active TB for a period of one year after 3HP treatment completion (*i.e.,* up to 16 months after enrollment) at clinic visits for routine HIV/AIDS care. TB screening will include symptom assessment using the WHO four-symptom TB screen administered at every visit. For patients with new or progressive TB symptoms a chest X-ray (**CXR**) and sputum for Xpert MTB/RIF and mycobacterial culture will be completed, as per routine WHO and NTLP recommended procedures. Sputum will be induced, if needed. All participants diagnosed with TB at any time point will have sputum submitted for culture and drug susceptibility testing on liquid media. Loss to follow-up will be defined as a participant not able to be contacted and does not return to clinic within the 12-month post treatment follow-up period.

The following *study-specific activities* will be performed by research staff after 3HP treatment is complete:

1. **Survey 3**: Research staff will administer a survey to document the reason for treatment discontinuation (completed therapy, adverse event/intolerance, other) and to assess satisfaction with TB preventive care received. Study staff will use patient telephone numbers and addresses to contact study participants. If returning to clinic is not possible, the survey may be conducted over the phone.

In addition, in-depth interviews will be conducted with selected trial participants and health service provides to identify barriers and facilitators to adoption and implementation of 3HP under each delivery strategy.

| **Table 2. Participant data collection and measurements.** | | | | |
| --- | --- | --- | --- | --- |
| **Procedure** | **Day 1** | **Weekly (DOT) or 6-week refill visit (SAT) during 3HP treatment**  **(Weeks 2-11)^a^** | **Post-3HP treatment** | |
|  |  |  | **Week 12^a^** | **Every 2-3 Months (Months 3-16)^a^** |
| **Baseline questionnaire**   - Demographic and clinical information | **X** |  |  |  |
| **Survey 1**   - Cost (to access clinic) - Knowledge/Beliefs/Support | **X** |  |  |  |
| **Survey 2^b^**   - Reasons for choice | **X** |  |  |  |
| **Time-and-Motion studies^c^** |  | **X** |  |  |
| **Patient costing survey^d^** |  | **X** |  |  |
| **3HP adherence assessment** |  | **X** | **X** |  |
| **Adverse events assessment** |  | **X** | **X** |  |
| **Active TB assessment^e^** |  | **X** | **X** | **X** |
| **3HP delivery process metrics** |  | **X** |  |  |
| **Survey 3**   - Satisfaction with care |  |  | **X** |  |
| **In-depth interview^f^** |  |  | **X** |  |

^a^ 3HP treatment may last up to 16 weeks

^b^ Patients randomized to choice arm only

^c^ Time-and-motion studies with selected participants in each arm at a randomly selected weekly (DOT) or 6-week refill (SAT) follow-up visit.

^d^ Selected participants from each arm.

^e^ Only if symptomatic, except at end of follow-up.

^f^ Selected participants from each arm.

## **Outcome Measures**

### **Primary Outcome (Effectiveness of delivery options)**

| **Outcome** | **Numerator** | **Denominator** |
| --- | --- | --- |
| Proportion accepting and completing 3HP treatment | Number who take at least 11 of 12 doses within 16 weeks of enrollment | Number randomized |

### **Secondary Outcomes (Quantitative)**

| **Outcome** | **Numerator** | **Denominator** |
| --- | --- | --- |
| **REACH** | | |
| Treatment acceptance | Number taking at least one dose of 3HP | Number randomized |
| **EFFECTIVENESS** | | |
| Treatment completion | Number who take at least 11 of 12 doses within 16 weeks of enrollment | Number taking at least one dose of 3HP |
| Cost-effectiveness (overall) | (Cost in Arm A – cost in Arm B) from societal perspective | (DALYs averted in Arm A – DALYs averted in Arm B) from societal perspective |
| Cost-effectiveness (health system) | As above, but from health system/provider perspective | As above, but from health system/provider perspective |
| Cost-effectiveness (patient) | As above, but from patient perspective | As above, but from patient perspective |
| Safety | Number for whom treatment discontinued due to adverse events or intolerance. | Number taking at least one dose of 3HP |
| Cumulative incidence of TB | Number diagnosed with TB during 16-month follow-up | Risk set* |
| **ADOPTION** | | |
| **All Participants** | | |
| Visit Cost Reimbursement | Number of visits for which reimbursement provided on same day | Total number of DOT/refill visits |
| Time to complete clinic visit | Mean and median number of minutes for each DOT/refill visit | -- |
| SMS or IVR phone call reminders delivered | Number of SMS or IVR phone call dosing or clinic visit reminders delivered | Total number of SMS reminders scheduled to be sent |
| SMS or IVR phone call reminders following missed appointments | Number of SMS or IVR phone call dosing or clinic missed appointment reminders delivered | Number of participant weeks with 3HP missed appointments |
| Screening for active TB | Number screened for active TB during DOT visits | Total number of DOT visits |
| Screening for side effects | Number screened for side effects during DOT visits | Total number of DOT visits |
| **Participants taking 3HP by SAT only** | | |
| Dosing confirmation via 99DOTS | Number of doses recorded by 99DOTS | Total number of scheduled doses (not including doses taken during clinic visits). |
| SMS or IVR phone call missed dose reminders delivered | Number of SMS or IVR phone call missed dose reminders delivered | Number of participant weeks with 3HP dosing not recorded by 99DOTS |
| SMS or IVR phone call weekly check-in delivered | Number of weekly SMS or IVR phone call check-ins delivered | Number of participant weeks taking 3HP (not including weeks where dosing is directly observed) |
| Follow up (phone calls or home visits) for negative response to weekly SMS or IVR phone call check-in | Number of follow-up calls and/or home visits completed following negative response to weekly SMS or IVR phone call check-in message | Number of participant weeks of negative response to weekly SMS or IVR phone call check-in messages |
| **IMPLEMENTATION** | | |
| Costs of preventive services | Mean total patient costs related to TB preventive care services | |
| Participant satisfaction | Mean score on PSQ questionnaire | -- |
| Barriers to 3HP delivery | Thematic interpretation of provider- and clinic-level barriers to care from provider focus group discussions | |
| Barriers to 3HP completion | Thematic interpretation of barriers to 3HP completion from patient interviews | |

***** Participants will be censored at the time of active TB diagnosis or treatment initiation, death, loss to follow-up (*i.e.,* unable to be contacted and have sputum collected between 10 and 14 months after 3HP treatment discontinuation or completion), or end of the 12-month post-treatment follow-up period

## **Statistical Considerations**

1. **Primary hypotheses:**
   1. The proportion who accept and complete 3HP will be highest among PLHIV randomized to the informed choice arm.
   2. The proportion of PLHIV who accept and complete 3HP can exceed 80% in a high HIV/TB burden setting.

**Sample size:** We estimated our sample size for the primary outcome (N=1656; 552/arm) based on a minimum clinically important difference of 10% in 3HP completion, comparing patient choice vs. DOT arms. Of note, the Centers for Disease Control and Prevention (**CDC**) TBTC iAdhere study of 3HP delivery strategies chose a 15% non-inferiority margin between DOT and SAT based on cost-effectiveness modeling in the US.^17^ We chose 10% to be more conservative because similar modeling studies have not been done in low-income settings, and because ours is not a non-inferiority design. To be maximally conservative, we applied a Bonferroni correction based on two independent comparisons (choice vs. DOT and choice vs. SAT). Assuming a two-sided alpha of 0.025, and 10% loss between consent and allocation, a sample size of 552 participants per arm (N=1656 total) is required to provide power of 0.90 to detect this difference (Table 3). This sample size will also give us power of 0.85 to detect a point estimate of at least 80% effectiveness in the patient choice arm, assuming a true effectiveness of 85%. If the true effectiveness rises to 86%, our power to show effectiveness >80% increases to 0.96. We calculated power using a continuity-corrected chi-squared statistic (or Fisher’s exact test) to evaluate the differences listed, assuming independent samples and a binomial distribution. Power was calculated using the PS - Power and Sample Size program (Dupont WD, Plummer WD: "Power and Sample Size Calculations: A Review and Computer Program", Controlled Clinical Trials 1990; 11:116-28).

**Table 3. Power to detect 10% difference**

| **Effectiveness** | | **Power** |
| --- | --- | --- |
| **DOT** | **Choice** |  |
| 70% | 80% | 0.90 |
| 75% | 85% | 0.94 |
| 80% | 90% | 0.98 |
| 85% | 95% | 0.998 |

1. **Data Analysis:**

***Primary analyses*** will involve assessment of all listed primary (effectiveness of delivery options) and secondary (reach, effectiveness, adoption, and implementation) outcomes.

- ***Primary outcome*** analysis will include: 1) unadjusted intent-to-treat comparisons of effectiveness between arms and 2) simple calculation (using the exact binomial confidence intervals) of the effectiveness of each delivery option, with a target of 80%. In these analyses, participants randomized to the choice arm will be treated separately from those randomized to either facilitated DOT or facilitated SAT; the proportion of those in the patient choice arm who choose DOT or SAT will be measured, but the primary outcome will consider all individuals in this arm as randomized to patient choice. Whether 80% acceptance and completion is achieved will be based on the lower bound of the Bonferroni-corrected 97.5% confidence interval exceeding 0.80 in any of the three arms. The intent-to-treat comparisons will use log-binomial regression to calculate prevalence ratios, and Fisher’s exact test to determine statistical significance (two-sided alpha of 0.025, including Bonferroni correction). Participants who decline to initiate 3HP after randomization will be counted as not accepting/completing treatment in the primary (intent-to-treat) analysis and will be followed over time for clinical outcomes.
- ***Secondary outcome*** analysis will include:
  1. ***Reach outcomes:*** Analysis will include 1) unadjusted comparisons across arms, 2) per protocol analysis of DOT versus SAT in the choice arm in case of crossover including analysis of outcomes among participants who crossed over against outcomes among those who stayed in their originally selected strategy if a sufficiently large number of patients cross over from DOT to SAT or vice versa and 3) simple calculation of the proportion of patients offered 3HP who accept treatment in each arm. We will also compare this outcome across key demographic variables including age, gender, CD4 stratum, and viral load suppression.
  2. ***Effectiveness outcomes***: Analysis of effectiveness outcomes will include the development of TB and the occurrence of adverse events analyzed with Chi-square tests, logistic regression, Kaplan-Meier curves, log-rank tests, and Cox regression methods as appropriate. Any signs or symptoms lasting >3 days duration (by patient self-report) during the previous month will be recorded and included in our analysis. We will also compare outcomes across key demographic variables including age and gender.
  3. ***Health economic outcomes***: Analysis of health economic outcomes will include 1) a *cost analysis* to estimate and compare the per-patient cost of delivering 3HP under each strategy and 2) a comparative *cost-utility analysis*, to identify the economically preferred option if there is a difference in effectiveness between arms (and the more effective option is also the more expensive one).
  4. ***Adoption outcomes****:* For each process metric, we will report the monthly proportion (95% Confidence Interval (**CI**)) or median (interquartile range (**IQR**)) to assess the implementation of intervention components in each arm overall and within sub-groups.
  5. ***Implementation outcomes:*** Analysis of implementation outcomes will include multivariable regression models to compare by study arm patient survey data including a) patient costs; b) scores on questions related to patient barriers to TB preventative care; and c) patient satisfaction; and also to identify factors independently associated with 3HP completion across study arms.

***Secondary analyses*** will include:

- Assessing our primary outcome using multivariable log-binomial models that adjust for the characteristics measured in the baseline survey (*e.g.,* ART status). If multivariable log-binomial models fail to converge, we will use a modified Poisson regression with robust estimates of variance.^28^ Comparison will be made to logistic regression models as well.
- Assessing our primary outcome to estimate a per protocol estimand that reflects the difference in treatment completion rates in patients that actually start treatment. Causal inference methods such as inverse probability weighting will be used to minimize bias in this estimator, full details will be given in the statistical analysis plan.
- Assessing our primary outcome to estimate a per protocol estimand for the comparison of DOT vs SAT irrespective of treatment arm in treatment competition rates (combining participants that choose to start on SAT with participants in the SAT arm and combining participants that choose to start on DOT with participants in the DOT arm). This analysis will be repeated as an ‘as treated’ analysis classification participants based on which delivery strategy they actually received for the majority of doses irrespective of which arm they were allocated to or which delivery method they started on in the choice arm. Causal inference methods such as inverse probability weighting will be used to minimize bias in this estimator, full details will be given in the statistical analysis plan.

# **Data Management**

Dr. Phillips (statistician) will oversee data management in conjunction with UCSF- and Uganda-based study coordinators using the National Institutes of Health (**NIH**)-recommended Research Electronic Data Capture (**REDCap**) software, password-protected and accessible only to research staff. All data collection forms will be entered into REDCap, with validation of data using range and consistency checks. Quality control procedures will include review of all study data collection forms for completeness and accuracy prior to data capture. Queries performed on the database will be reviewed, verified and corrected on a twice-monthly basis. The UCSF study coordinator will visit Uganda 2-3 times a year and review a random sample of forms and primary data sources for quality assurance.

# **Ethical Considerations**

- 1. **Potential risks to participants:** The primary risk to participants is that of medication-induced adverse events (due to isoniazid or rifapentine), a risk that is greatly outweighed in this population by the potential benefit of prevented active TB among participants who complete treatment. 3HP is an approved treatment regimen for TB preventive therapy that has a lower rate of side effects than the most commonly used regimen (isoniazid daily for 9 months). In the PREVENT TB trial, major adverse events (Grade 3 or 4) occurred in 1.6% of patients.^10^ Overall, 4.9% of patients discontinued treatment due to adverse effects, including 2.9% due to hypersensitivity reactions. Hypersensitivity reactions may include a flulike syndrome (*e.g.*, fever, chills, headaches, dizziness, musculoskeletal pain), thrombocytopenia, shortness of breath or other signs and symptoms including wheezing, acute bronchospasm, urticaria, petechiae, purpura, pruritus, conjunctivitis, angioedema, hypotension or shock. Other side effects, including hepatotoxicity, are uncommon with once-weekly dosing but will also be assessed at every clinic visit. Importantly, rifapentine given once weekly has also been shown to not have any negative effects on the efficacy of efavirenz-based ART regimens.

In addition to the risk of medication-induced adverse events, there is also a risk of privacy loss, specifically as patients will be attending pharmacy-only visits that may identify them as participants in the trial. However stable patients on ART also undergo pharmacy-only visits at the Mulago ISS clinic. Other risks – such as breach of confidentiality during data storage and upload, and loss of time/inconvenience during the interview process – are not anticipated to be any greater in this trial than in other randomized trials or observational studies of PLHIV in sub-Saharan Africa. The primary research risks for clinicians related to the qualitative studies are punitive actions by the employer in response to the information they provide for research.

- 1. **Protection against risk:** The trial will be submitted for approval to the Research Ethics Committees of the University of California San Francisco and the Makerere University School of Public Health Higher Degrees Research and Ethics Committee (HDREC), and to the Uganda National Council for Science and Technology. It is registered with the U.S. National Institutes of Health’s ClinicalTrials.gov as a Phase 4 clinical trial. All study staff will be required to have completed Good Clinical Practice (**GCP**) training and human subjects research training. The Directors at Mulago ISS clinic will be asked to sign a Memorandum of Understanding agreeing that their health center participate in the trial and agreeing not to introduce any new TB evaluation interventions during the time period of the trial without informing trial staff.

To minimize risks to autonomy for patients and providers who participate in the study, research staff will be carefully trained in how to administer the consent form, with attention given to the background and principles of research ethics. Care will be taken to protect the confidentiality of participants’ HIV status during enrollment and collection of data. We will obtain written consent from all patients who participate in the study. We will obtain verbal consent using a script for participation in patient interviews. A member of the research team will review the information sheet in detail with each participant and answer any questions. They will emphasize that participation is voluntary, that consent can be withdrawn at any time, and that a participant can refuse to answer any specific question (see attached Verbal Consent form). The verbal consent process will be audio recorded for documentation. Written consent will not be obtained because no participant identifiers will be collected and the consent document would be the only record linking the participant to the study. Written informed consent will be obtained from providers who participate in direct observation (*i.e.,* time-motion) studies and/or focus group discussions/interviews. The consent forms, which will be approved by IRBs in the U.S. and Uganda, will be translated from English into the local languages, and back-translated into English as required by IRBs to be sure that no significant language or concepts are lost in translation.

Study procedures will be under regular oversight by the institutional review boards at Makerere University and the University of California, San Francisco, as well as by the Uganda National Council for Sciences and Technology, a government body. In addition, we will liaise with the Ugandan IRBs to establish a community-advisory board to discuss the goals and methods of the planned interventions to seek advice in tailoring the program, and assistance in publicizing our activities.

- 1. **Data protection:** All patient-identifiable data will be stored in locked or password-protected areas accessible only to study personnel. Personal data stored in mobile service-provider databases will only include personal phone numbers (which do not require owners to provide their names or other identifying information when purchased and are therefore not routinely recorded in public directories). No other identifying information will be included in text messages or IVR phone calls. All messages will be generic in nature and not include specific disease information, test results, or clinic details. To protect participant confidentiality, all study staff and clinicians accessing the 99DOTS server will be required to have a unique login and password.

All surveys/interview transcripts will be kept in a secure, locked file cabinet or password-protected database. Cost survey data will be entered into the REDCap software. The REDCap database will be maintained on a password-protected server accessible only to study staff. Audio recordings of interviews will be destroyed after transcription. Transcripts will be checked to remove any names or other participant identifiers prior to being stored as data files in the REDCap database.

- 1. **Potential benefits of the study to participants and society:** Preventive therapy is known to reduce the risk of TB by 30-50% among PLHIV. Although short-course therapy with 3HP is less well studied in high burden settings, it has been shown to have equal or better efficacy to IPT in clinical trials, and we therefore anticipate that participants will benefit from reduced incidence of active TB and associated morbidity and mortality. Participants will also benefit from regular screening for active TB, which could contribute to earlier diagnosis and treatment than would have occurred in routine clinical care. Potential benefits to society include identification of strategies to improve delivery of 3HP in the context of HIV/AIDS care in low-income high TB/HIV burden countries. If successful, the delivery strategies could potentially be scaled up to improve delivery of TB preventive therapy in similar settings.
  2. **Data and safety monitoring plan:** The principal investigators of the study will have overall responsibility for monitoring data collection, study progress and safety in consultation with an Independent Steering Committee.
     1. Data collection: The Data Manager will generate bi-weekly reports to check data quality, including completeness and out of range responses. The reports will be reviewed by the PIs and in-country staff will be responsible for resolving queries.
     2. Study progress: The Steering Committee will review the research protocol and plans for safety and data monitoring and make recommendations for any changes to the PIs. The Data Manager will generate bi-weekly enrollment reports to track the progress of recruitment, enrollment, and retention. The PIs will review the reports and make changes as needed. The Steering Committee will also review quarterly reports on study progress, adverse events, breaches of confidentiality, AND unanticipated problems involving risk to participants or others. No interim safety or efficacy analyses are planned, as this is an implementation trial of an approved TB preventive therapy regimen.
     3. Safety: Because this is an implementation trial, adverse events related to TB preventive therapy will be monitored as they would in routine practice/care. Briefly, for participants taking 3HP by DOT and attending weekly clinic visits, clinicians will administer a side effect screen, as per routine procedures. Participants taking 3HP by SAT will receive a weekly check-in via SMS or IVR phone call asking if they are well, with a follow up call from clinicians if necessary, to reflect standard screening procedures. Routine clinicians will make all decisions to interrupt or discontinue treatment and study staff will review patient charts to document reasons for interruption or discontinuation. As required by the Uganda National Drug (NDA) Authority, reasons for treatment interruption or discontinuation related to adverse events will be reported to the NDA. In addition, if any hospitalizations or deaths that clinicians suspect could be related to 3HP will be reported to the NDA and to the local and UCSF IRBs. Participant safety data will be part of the quarterly reports sent to the Steering Committee, who will review reports and make recommendations to the PIs of any need to enhance safety monitoring plans beyond what would occur in routine care and/or to discontinue the study.

- 1. **Dissemination:** The trial results will be communicated to stakeholders through dissemination meetings and to participating health centers using language-appropriate information sheets. Investigators will present results at relevant conferences, and submit manuscript(s) to peer-reviewed journals. Public access to the participant-level dataset of main trial results and statistical code will be made available.

1. **Abbreviations**

3HP Three-month regimen weekly isoniazid and rifapentine

ART Anti-retroviral therapy

CDC Centers for Disease Control and Prevention

CHW Community Health Worker

CI Confidence Interval

CTCAE Common Terminology Criteria for Adverse Events

DALY Disability-Adjusted Life Year

DOT Directly Observed Therapy

GCP Good Clinical Practice

GNI Gross National Income

IDI In depth interviews

INH Isoniazid

IRB Institutional Review Board

IQR Interquartile Range

IVR Interactive Voice Response

KII Key Informant Interviews

LTBI Latent Tuberculosis Infection

MJAP Makerere University Joint AIDS Program

NDA National Drug Authority

NHLBI National Heart, Lung, and Blood Institute

NIH National Institutes of Health

NTLP National Tuberculosis and Leprosy Program

PACTR Pan African Clinical Trials Registry

PEPFAR US President’s Emergency Fund for AIDS Relief

PLHIV People Living with HIV

REDCap Research Electronic Data Capture

RPT Rifapentine

SAT Self-administered Therapy

SDM Shared Decision Making

SMS Short Message Service

TB Tuberculosis

UCSF University of California San Francisco

WHO World Health Organization

1. **References**

1. Group TAS. A Trial of Early Antiretrovirals and Isoniazid Preventive Therapy in Africa. *N Engl J Med.* 2015.

2. Dye C, Glaziou P, Floyd K, Raviglione M. Prospects for tuberculosis elimination. *Annu Rev Public Health.* 2013;34:271-286.

3. Getahun H, Granich R, Sculier D, et al. Implementation of isoniazid preventive therapy for people living with HIV worldwide: barriers and solutions. *AIDS.* 2010;24 Suppl 5:S57-65.

4. Motsamai OI. *IPT Botswana Experience - Ministry of Health Botswana.*: World Health Organization: Meeting Presentation. Addis Ababa, Ethiopia.;2011.

5. WHO. *Report of a "lessons learnt" workshop on the six ProTEST pilot projects in Malawi, South Africa and Zambia.* Geneva, Switzerland: World Health Organization: Stop TB and HIV/AIDS Departments;2004.

6. Namuwenge PM, Mukonzo JK, Kiwanuka N, et al. Loss to follow up from isoniazid preventive therapy among adults attending HIV voluntary counseling and testing sites in Uganda. *Trans R Soc Trop Med Hyg.* 2012;106(2):84-89.

7. WHO. Guidelines for intensified tuberculosis case-finding and isoniazid preventive therapy for people living with HIV in resource-constrained settings. In. Geneva, Switzerland: WHO Press; 2011.

8. WHO. *Global Tuberculosis Report 2016.* Geneva, Switzerland. Available at: <http://www.who.int/tb/publications/global_report/en/> 2016.

9. Martinson NA, Barnes GL, Moulton LH, et al. New regimens to prevent tuberculosis in adults with HIV infection. *N Engl J Med.* 2011;365(1):11-20.

10. Sterling TR, Villarino ME, Borisov AS, et al. Three months of rifapentine and isoniazid for latent tuberculosis infection. *N Engl J Med.* 2011;365(23):2155-2166.

11. Podany AT, Bao Y, Swindells S, et al. Efavirenz Pharmacokinetics and Pharmacodynamics in HIV-Infected Persons Receiving Rifapentine and Isoniazid for Tuberculosis Prevention. *Clinical infectious diseases : an official publication of the Infectious Diseases Society of America.* 2015.

12. Farenc C, Doroumian S, Cantalloube C, et al. Rifapentine Once-Weekly Dosing Effect on Efavirenz, Emtricitabine and Tenofovir Pharmacokinetics. Conference on Retroviruses and Opportunistic Infections; 2014; Boston, MA, USA.

13. Weiner M, Egelund EF, Engle M, et al. Pharmacokinetic interaction of rifapentine and raltegravir in healthy volunteers. *J Antimicrob Chemother.* 2014;69(4):1079-1085.

14. Dooley KE CG, Savic RM, Gupte A, Marzinke MA, Zhang N, Edward V, Wolf L, Sebe M, Likoti M, Fyvie M, Shibambo I, Beattie T,Chaisson RE. Safety & PK of weekly rifapentine/isoniazid (3HP) in adults with HIV on dolutegravir. CROI; 4-7 March 2019, 2019; Seattle.

15. Swindells S, Ramchandani R, Gupta A, et al. One Month of Rifapentine plus Isoniazid to Prevent HIV-Related Tuberculosis. *N Engl J Med.* 2019;380(11):1001-1011.

16. (SANAC) SANAC. National Strategic Plan on HIV, STIs, and TB 2017-2021. *Available at:* [*http://nspsanacorgza/*](http://nspsanacorgza/)*.* 2017.

17. Belknap R, Holland D, Feng PJ, et al. Self-administered Versus Directly Observed Once-Weekly Isoniazid and Rifapentine Treatment of Latent Tuberculosis Infection: A Randomized Trial. *Ann Intern Med.* 2017;167(10):689-697.

18. Liu X, Blaschke T, Thomas B, et al. Usability of a Medication Event Reminder Monitor System (MERM) by Providers and Patients to Improve Adherence in the Management of Tuberculosis. *Int J Environ Res Public Health.* 2017;14(10).

19. America CoQoHCi. *Crossing the Quality Chasm: A New Health System for the 21st Century.* Washington, DC: Institute of Medicine;2001.

20. Charles C, Gafni A, Whelan T. Shared decision-making in the medical encounter: what does it mean? (or it takes at least two to tango). *Soc Sci Med.* 1997;44(5):681-692.

21. Joosten EA, DeFuentes-Merillas L, de Weert GH, Sensky T, van der Staak CP, de Jong CA. Systematic review of the effects of shared decision-making on patient satisfaction, treatment adherence and health status. *Psychother Psychosom.* 2008;77(4):219-226.

22. Stacey D, Bennett CL, Barry MJ, et al. Decision aids for people facing health treatment or screening decisions. *Cochrane Database Syst Rev.* 2011(10):CD001431.

23. Epstein RM, Franks P, Fiscella K, et al. Measuring patient-centered communication in patient-physician consultations: theoretical and practical issues. *Soc Sci Med.* 2005;61(7):1516-1528.

24. Durand MA, Carpenter L, Dolan H, et al. Do interventions designed to support shared decision-making reduce health inequalities? A systematic review and meta-analysis. *PLoS One.* 2014;9(4):e94670.

25. O'Connor AM, Rostom A, Fiset V, et al. Decision aids for patients facing health treatment or screening decisions: systematic review. *BMJ.* 1999;319(7212):731-734.

26. Consolidated Guidelines for the Prevention and Treatment of HIV and AIDS in Uganda. In: Health Mo, ed. Uganda2020.

27. *Tuberculosis Patient Cost Surveys: A Handbook.* Geneva: World Health Organization;2017.

28. Spiegelman D, Hertzmark E. Easy SAS calculations for risk or prevalence ratios and differences. *Am J Epidemiol.* 2005;162(3):199-200.
