# Supplementary material for: Completion of isoniazid–rifapentine (3HP) for tuberculosis prevention among people living with HIV: Interim analysis of a hybrid type 3 effectiveness–implementation randomized trial
Source: PLoS Med. 2021 Dec 16;18(12):e1003875. doi: 10.1371/journal.pmed.1003875 (PMC8726462; doi:10.1371/journal.pmed.1003875)
Supplement: S1 Text — TB, tuberculosis. (DOCX) [file pmed.1003875.s006.docx]

| **Side effects/toxicity screening checklist** | |
| --- | --- |
| **Does patient have any side effects or signs of toxicity?** | Select Yes or No. If yes, further questions regarding specific side effects being experienced will appear. |
| **Side effects: Check if present.** | Yes or No can be selected for the following side effects displayed in a matrix:   1. Loss of appetite 2. Nausea or vomiting 3. Yellow eyes or skin 4. Abdominal pain 5. Diarrhea 6. Rash/Hives 7. Fever or chills 8. Dizziness/fainting 9. Numbness or tingling 10. Joint pain 11. Headache 12. Itching 13. Weakness 14. Other |
| **TB screening checklist** | |
| **Patient coughing of any duration?** | Yes or No can be selected for the following symptoms of TB. |
| **Patient fever ≥2 weeks?** |  |
| **Patient noticeable weight loss (more than 3kg)?** |  |
| **Patient excessive night sweats ≥3 weeks?** |  |
| **Referred to clinician for potential adverse event?** |  |
