# Supplementary material for: Completion of isoniazid–rifapentine (3HP) for tuberculosis prevention among people living with HIV: Interim analysis of a hybrid type 3 effectiveness–implementation randomized trial
Source: PLoS Med. 2021 Dec 16;18(12):e1003875. doi: 10.1371/journal.pmed.1003875 (PMC8726462; doi:10.1371/journal.pmed.1003875)
Supplement: S2 Text — AE, adverse event. (DOCX) [file pmed.1003875.s007.docx]

**Serious Adverse Events (SAE) Summary:**

| **Study ID** | **SAE Description** | **Seriousness Criteria** | **Severity** | **Relation to 3HP** | **SAE Outcome** |
| --- | --- | --- | --- | --- | --- |
| 2589 | Generalized, persistent pruritus | Significant and persistent incapacity | Severe | Possibly | Recovered |
| 2004 | Venous Thromboembolism, right leg | Significant and persistent incapacity | Severe | Possibly | Recovered |
| 2083 | Flu-like Syndrome and peripheral neuropathy | Significant and persistent incapacity | Moderate | Probably | Recovered with sequelae |
| 1798 | Pulmonary embolism | Significant and persistent incapacity | Severe | Possibly | Recovered with sequelae |
| 2637 | Generalized pruritus | Significant and persistent incapacity | Moderate | Probably | Recovered |
| 2525 | Persistent pruritic rash | Significant and persistent incapacity | Moderate | Probably | Recovered |
| 2007 | Persistent pruritic desquamative rash | Significant and persistent incapacity | Severe | Probably | Recovered |
| 2324 | Acute liver injury | Life threatening, Requires in-patient hospitalization, Significant and persistent incapacity, Important medical condition | Severe | Probably | Recovered |

**Concomitant medication with known 3HP interaction:**

| **Study ID** | **SAE Description** | **Seriousness Criteria** | **Severity** | **Relation to 3HP** | **Concomitant Medication** | **Outcome** |
| --- | --- | --- | --- | --- | --- | --- |
| 1865 | Erosive gastritis | Significant and persistent incapacity | Severe | Not related | Clarithromycin, Omeprazole, Domperidone | Recovering |

**Seriousness Criteria - description:**

A serious adverse event is an event, which:

|  | Results in death |
| --- | --- |
|  | Is life threatening |
|  | Requires in patient hospitalization or prolongation of existing hospitalization |
|  | Results in significant and persistent incapacity |
|  | Is a congenital anomaly or birth defect |
|  | Is an important medical condition in the opinion of the investigator. |

**Severity Criteria – description:**

| **Mild** | The subject is aware of the event or symptom, but the event or symptom is easily tolerated (e.g. no reduction in daily activities is required) |
| --- | --- |
| **Moderate** | The subject is aware of the event or symptom, but the event or symptom is easily tolerated (e.g. no reduction in daily activities is required) |
| **Severe** | Significant impairment of functioning: the subject is unable to carry out usual activities and/or the subject’s life is at risk from the event. |
| **Life-threatening** | The subject is at risk of death at the time of the adverse event; it does not refer to an AE that hypothetically might have caused death if more severe. |
| **Death** | The subject dies as a result of the adverse event |

**Relatedness of SAE to intervention – description:**

| Definitely | When the event is directly caused by the intervention. |
| --- | --- |
| Probably | When the event is most likely explained by the research intervention but when definite proof of causality is not evident. |
| Possibly | When explanation for event is equally due to research intervention or other cause. |
| Unlikely | When the event is more likely explained by another cause. |
| Not related | When the event is clearly due to another cause. |
