# Supplementary material for: Completion of isoniazid–rifapentine (3HP) for tuberculosis prevention among people living with HIV: Interim analysis of a hybrid type 3 effectiveness–implementation randomized trial
Source: PLoS Med. 2021 Dec 16;18(12):e1003875. doi: 10.1371/journal.pmed.1003875 (PMC8726462; doi:10.1371/journal.pmed.1003875)
Supplement: Attachment — Submitted filename: Review notes.docx [file pmed.1003875.s008.docx]

This manuscript details an interim analysis of the ongoing *3HP Options Trial* in Uganda*,* a randomized trial of DOT vs. SAT vs. SAT+DAT that is evaluating acceptance and completion rates across these three treatment strategies for TPT in PLHIV. Considering the need for more evidence for acceptance and adherence of these optimized strategies in the context of routine HIV care in sub-Saharan Africa, this manuscript will directly and substantially support the WHO’s recommendation for using 3HP as an option for TPT in this setting.

The authors describe an inference analysis, estimating that at least one optimized treatment strategy would lead to ≥80% acceptance and completion of 3HP. Following their analysis, the authors demonstrated that all three strategies had a very high probability of exceeding 80% acceptance and treatment completion, effectively supporting that all three optimized strategies are highly effective in a sub-Saharan Africa context in Uganda.

The findings of this analysis are in context of previous literature on the topic, and the data analysis clearly supports the findings presented.

This manuscript details an interim analysis of an ongoing randomized trial and details of trial design have been previously published and are accessible via PubMed.

The overall study conforms to CONSORT guidelines for a randomized trial, and the methodology for the analysis to be reproduced.

Overall, the manuscript is well written, is accessible to non-specialists, and the findings will add to the body of evidence supporting high acceptability and completion rates for 3HP for TPT, using both DOT and SAT approaches, in the context of routine HIV care in sub-Saharan Africa.

Line-by-line minor comments, questions:

Line 90 – consider emphasizing that the Belknap study demonstrated that SAT was noninferior to DOT, but only specifically in the United States component of this study (further supports the authors’ argument that evidence on 3HP acceptability and completion rates from studies conducted outside the United States, especially in SSA, is needed.

114 – is the digital adherence technology (DAT) text based? Consider adding explanation on what the participant was required to do with DAT. IVR is clearly explained in the manuscript.

129 – per the primary outcome: “the proportion of randomized participants who accept 129 3HP treatment and complete at least 11 of the 12 weekly doses within 16 weeks of enrollment.” Did any participants who reached this outcome have a treatment interruption of 4 weeks before resuming weekly dosing and completing 3HP? I ask this as professional opinion can vary on the acceptable maximum treatment interruption allowed before a patient must restart 3HP (in my experience, between 1 and 4 weeks). Consider including the range of treatment interruption (in days) that was observed by this analysis for individuals who did successfully complete 3HP.

150 (Figure 1) – one ‘did not meet inclusion criteria’ in figure 1 states: “resistance to isoniazid (INH) or rifapentine (RPT) (n=2)”. How was this determined/known? Were these individuals previously treated for active TB disease and now qualified for TPT? Seems that would not be the case as another ‘did not meet inclusion criteria’ is “prior treatment for TB/preventive TB”. Please clarify.

179 – considering describing most common adverse event(s) that led to 1.7% of participants discontinuing treatment.

182 – unless it represents an increased risk for identifying the participant, consider including the name of the medication with a known 3HP interaction

Overall, a well written manuscript. My recommendation is ‘accept with minor revision’.

Clay Roscoe, MD MSc

HIV Services, Family Medicine Residency of Idaho, Boise, Idaho, USA

CMO, Sedia Biosciences, Beaverton, OR, USA
