# Supplementary material for: Completion of isoniazid–rifapentine (3HP) for tuberculosis prevention among people living with HIV: Interim analysis of a hybrid type 3 effectiveness–implementation randomized trial
Source: PLoS Med. 2021 Dec 16;18(12):e1003875. doi: 10.1371/journal.pmed.1003875 (PMC8726462; doi:10.1371/journal.pmed.1003875)
Supplement: Attachment — Submitted filename: 3HP Options Trial_Interim Analysis_Review response_final.docx [file pmed.1003875.s009.docx]

Dear Dr. Davidson,

Thank you for your comments on our manuscript titled "Completion of Isoniazid-Rifapentine (3HP) for tuberculosis (TB) prevention among people living with HIV (PLHIV): interim analysis of the 3HP Options Trial." Per your comments and those of the reviewers, we have added additional details and edits to our attached revised manuscript. With these additions, we believe the manuscript more clearly demonstrates important details of the trial implementation and the goals of this interim analysis and will be of value to the *PLOS Medicine* readership.

Please note that all line numbers in the responses below refer to line numbers in the clean version of the resubmitted manuscript, and all references included in excerpts from the revised manuscript that are included in this review response can be found at the end of this document.

**Editor comments**

In revising the manuscript for further consideration, your revisions should address the specific points made by each reviewer and the editors. Please also check the guidelines for revised papers at [https://urldefense.com/v3/__http://journals.plos.org/plosmedicine/s/revising-your-manuscript__;!!LQC6Cpwp!5BjwNxNBY5EdUc-sh1LzmXNv4EqM3isSCHmHsabmfzIX_F3SK2aiDfBSWNJuYCfWV3IG1gd0$](https://urldefense.com/v3/__http:/journals.plos.org/plosmedicine/s/revising-your-manuscript__;!!LQC6Cpwp!5BjwNxNBY5EdUc-sh1LzmXNv4EqM3isSCHmHsabmfzIX_F3SK2aiDfBSWNJuYCfWV3IG1gd0$)  for any that apply to your paper. In your rebuttal letter you should indicate your response to the reviewers' and editors' comments, the changes you have made in the manuscript, and include either an excerpt of the revised text or the location (eg: page and line number) where each change can be found. Please submit a clean version of the paper as the main article file; a version with changes marked should be uploaded as a marked up manuscript.

**Response**: We have done as requested. Please find below responses to specific comments. We have submitted clean and marked up versions of the revised manuscript.

1. *“As a general comment, please consider toning down your statement of generalisability to other programmatic settings (considering the single country setting and investment that would be required to make the interventions more widely available).”*

**Response**: Thank you for this comment. As suggested, we have toned this down and added it as a limitation to the Discussion section:

“Second, the trial was conducted at a single HIV/AIDS clinic in Uganda. However, the setting is similar to PEPFAR-funded HIV/AIDS clinics in other countries in sub-Saharan Africa, where similar barriers to completion of TB preventive therapy have been widely reported [1-8]. Third, some of the facilitation strategies evaluated as part of the 3HP Options trial are not likely to be part of usual care at most HIV/AIDS clinics in sub-Saharan Africa and would require additional funding to implement as part of 3HP scale-up.”

*2. “Please revise your title to indicate that this is an implementation science study. Your title should begin with main concept if possible. Please place the study design (e.g. "a randomized hybrid type 3 effectiveness-implementation trial”, or similar) design in the subtitle (i.e., after the colon).”*

**Response**: We have revised the title as suggested: “Completion of isoniazid-rifapentine (3HP) for tuberculosis prevention among people with HIV: interim analysis of a hybrid type 3 effectiveness-implementation randomized trial”

*3. “Please report your abstract according to CONSORT for abstracts, following the PLOS Medicine abstract structure (Background, Methods and Findings, Conclusions*

[*https://urldefense.com/v3/__http://www.consort-statement.org/extensions?ContentWidgetId=562v__;!!LQC6Cpwp!5BjwNxNBY5EdUc-sh1LzmXNv4EqM3isSCHmHsabmfzIX_F3SK2aiDfBSWNJuYCfWV_5C861v$*](https://urldefense.com/v3/__http:/www.consort-statement.org/extensions?ContentWidgetId=562v__;!!LQC6Cpwp!5BjwNxNBY5EdUc-sh1LzmXNv4EqM3isSCHmHsabmfzIX_F3SK2aiDfBSWNJuYCfWV_5C861v$)*”*

*Abstract:*

- *Please combine the Methods and Findings sections into one section, “Methods and findings”.*
- *Please specify who was blinded to the intervention arms.*
- *Please indicates the dates during which study enrollment and follow up occurred.*
- *Please include the main outcome measures.*
- *In the last sentence of the Abstract Methods and Findings section, please describe the main limitation(s) of the study's methodology.*

**Response**: We have made each of the suggested changes to the abstract.

*4. “At this stage, we ask that you include a short, non-technical Author Summary of your research to make findings accessible to a wide audience that includes both scientists and non-scientists. The Author Summary should immediately follow the Abstract in your revised manuscript. This text is subject to editorial change and should be distinct from the scientific abstract. Please see our author guidelines for more information:*

[*https://urldefense.com/v3/__https://journals.plos.org/plosmedicine/s/revising-your-manuscript*loc-author-summary__;Iw!!LQC6Cpwp!5BjwNxNBY5EdUc-sh1LzmXNv4EqM3isSCHmHsabmfzIX_F3SK2aiDfBSWNJuYCfWVzByFoX_$*](https://urldefense.com/v3/__https://journals.plos.org/plosmedicine/s/revising-your-manuscript*loc-author-summary__;Iw!!LQC6Cpwp!5BjwNxNBY5EdUc-sh1LzmXNv4EqM3isSCHmHsabmfzIX_F3SK2aiDfBSWNJuYCfWVzByFoX_$)*”*

**Response**: We have included an author summary with our resubmission on the page following the abstract in our revised manuscript.

*5. “Please update the section heading ‘Background’ to ‘Introduction’”*

**Response**: We have made this change to the revised manuscript.

*6. “Please provide a definition of hybrid trials and your justification for using a hybrid type 3 effectiveness implementation design, as some readers may not be familiar with the term”*

**Response**: Thank you for the opportunity to clarify. We have provided an explanation and relevant references under “Study design and participants”:

“Hybrid effectiveness-implementation trials assess both effectiveness and implementation outcomes, with Type I designs having a greater focus on intervention effectiveness and Type 3 designs having a greater focus on implementation strategies and outcomes [9].”

*7. “Please differentiate your clinical and implementation outcomes. It will be helpful to specify implementation outcomes using standard Implementation Science terminology such as e.g., acceptability, sustainability. This tool may be of use* [*https://urldefense.com/v3/__https://cpb-us-w2.wpmucdn.com/sites.wustl.edu/dist/6/786/files/2017/08/DIRC-implementation-outcomes-tool-dg_7-27-17_ab-27xbrka.pdf__;!!LQC6Cpwp!5BjwNxNBY5EdUc-sh1LzmXNv4EqM3isSCHmHsabmfzIX_F3SK2aiDfBSWNJuYCfWV0L2EpAM$*](https://urldefense.com/v3/__https:/cpb-us-w2.wpmucdn.com/sites.wustl.edu/dist/6/786/files/2017/08/DIRC-implementation-outcomes-tool-dg_7-27-17_ab-27xbrka.pdf__;!!LQC6Cpwp!5BjwNxNBY5EdUc-sh1LzmXNv4EqM3isSCHmHsabmfzIX_F3SK2aiDfBSWNJuYCfWV0L2EpAM$)*”*

**Response**: Thank you for the opportunity to clarify. While the overall 3HP Options Trial itself is a type 3 hybrid effectiveness-implementation trial, in this interim analysis we are primarily reporting on a single outcome which reflects the reach (3HP acceptance) and fidelity (3HP completion) of the intervention (*i.e.,* 3HP). Other implementation and effectiveness outcomes, which are detailed in our published trial protocol, were not included as part of this interim analysis. Nevertheless, we have clarified in the manuscript that we developed study outcomes according to the RE-AIM evaluation framework and included implementation science language indicating that the primary outcome assessed both intervention reach (acceptance) and fidelity (treatment completion):

“Trial outcomes were developed using the RE-AIM evaluation framework[10]. The primary outcome, which aims to assess both intervention reach (acceptance) and fidelity (treatment completion), is the proportion of randomized participants who accept 3HP treatment and complete at least 11 of the 12 weekly doses within 16 weeks of enrollment [11-13].”

*8. “Please clearly specify and report your implementation strategies. Consider using the guidelines published by Proctor et al. to improve your reporting:  Proctor EK, Powell BJ, McMillen JC: Implementation strategies: recommendations for specifying and reporting. Implement Sci 2013, 8:139”*

**Response**: We note that details of the three 3HP delivery strategies are provided in the published protocol paper. We originally included minimal details on these strategies as our interim analysis focuses on outcomes aggregated across trial arms rather than comparisons between trial arms (*i.e.,* between the three delivery strategies). However, based on the comment from the editor as well as several reviewers, we have provided additional detail while also more clearly referring to the prior published protocol paper in lines 182-201 on page 9 of the clean revised manuscript.

*9. “Please provide the names of the institutional review boards that provided ethical approval.”*

**Response**: Thank you, this is now included under “Study design and participants”, and reads as follows:

**“**The trial was approved by institutional review boards at Makerere University School of Public Health Higher Degrees, Research and Ethics Committee, the University of California San Francisco Human Research Protection Program, and by the Uganda National Council for Science and Technology.”

*10. “Please define the length of follow up (eg, in mean, SD, and range).”*

**Response**: We included the average length of follow up and range of days to complete among those completing treatment in the Results section:

“The mean length of follow-up among those completing treatment was 78.4 days (standard deviation: 3.5 days) and ranged from 72 days to 109 days. The longest treatment interruption among any participant reaching the primary outcome was 28 days.”

*11. “Citations should be in square brackets, please update throughout.”*

**Response**: Thank you, this has been corrected throughout the edited manuscript.

*12. “Please complete the CONSORT checklist and ensure that all components of CONSORT are present in the manuscript. When completing the checklist, please use section and paragraph numbers, rather than page numbers.*

**Response**: Thank you, this is included with our resubmission. We would like to note that the full checklist will be more appropriate for the manuscript describing the full trial design details and final trial results. We have therefore indicated “NA” (Not Applicable) and other clarifying notes for certain items on the checklist that are not relevant for this interim analysis.

*13. “In addition to the CONSORT checklist please ensure that your implementation research is reported according to Standards for Reporting Implementation Studies statement (STARI). The STARI guidelines can be found here:* [*https://urldefense.com/v3/__https://www.equator-network.org/reporting-guidelines/stari-statement/__;!!LQC6Cpwp!5BjwNxNBY5EdUc-sh1LzmXNv4EqM3isSCHmHsabmfzIX_F3SK2aiDfBSWNJuYCfWV2lxVegX$*](https://urldefense.com/v3/__https:/www.equator-network.org/reporting-guidelines/stari-statement/__;!!LQC6Cpwp!5BjwNxNBY5EdUc-sh1LzmXNv4EqM3isSCHmHsabmfzIX_F3SK2aiDfBSWNJuYCfWV2lxVegX$)*”*

**Response:** Thank you, the STARI checklist has been included with our resubmission. However, we would like to note that the full checklist will be more appropriate for the manuscript describing the final trial results that compare outcomes between the 3HP delivery (*i.e.,* implementation) strategies. We have therefore indicated “NA” (Not Applicable) for certain items on the checklist that are not relevant for this interim analysis.

*In accordance with ICMJE requirements, PLOS Medicine requires prospective, public registration of a data sharing plan (as part of mandatory clinical trials registration) for all clinical trials that began enrollment on or after January 1, 2019.”*

**Response**: Thank you, these details are included with our trial registration, which can be found under “Individual Participant Data (IPD) Sharing Statement following this link:

<https://clinicaltrials.gov/ct2/show/NCT03934931>

*14. Please include the study protocol document and analysis plan, with any amendments, as Supporting Information to be published with the manuscript if accepted.”*

**Response**: We have included the study protocol and the interim analysis plan along with our resubmission.

*15. To enhance the reproducibility of your results, we recommend that you deposit your laboratory protocols in protocols.io, where a protocol can be assigned its own identifier (DOI) such that it can be cited independently in the future. Additionally, PLOS ONE offers an option to publish peer-reviewed clinical study protocols. Read more information on sharing protocols at*

[https://urldefense.com/v3/__https://plos.org/protocols?utm_medium=editorial-email&utm_source=authorletters&utm_campaign=protocols__;!!LQC6Cpwp!5BjwNxNBY5EdUc-sh1LzmXNv4EqM3isSCHmHsabmfzIX_F3SK2aiDfBSWNJuYCfWVyR5lfjc$](https://urldefense.com/v3/__https:/plos.org/protocols?utm_medium=editorial-email&utm_source=authorletters&utm_campaign=protocols__;!!LQC6Cpwp!5BjwNxNBY5EdUc-sh1LzmXNv4EqM3isSCHmHsabmfzIX_F3SK2aiDfBSWNJuYCfWVyR5lfjc$)

**Response**: Thank you for this opportunity, however the trial protocol has already been published and is referenced in the manuscript (reference #14 in the manuscript document).

**Reviewer Comments**

***Reviewer #1:***

*Completion of Isoniazid-Rifapentine (3HP) for tuberculosis (TB) prevention among people living with HIV (PLHIV): interim analysis of the 3HP Options Trial" describes interim results for a randomized trial involving three arms, each investigating different therapy strategies: facilitated directly observed therapy (DOT), facilitated self-administered therapy (SAT), or informed choice between DOT and SAT. From 479 trial participants (approximately equally divided between the arms) having exited the treatment period, it was found that some 93% had completed the 3HP treatment, under the definition of having at least 11 of 12 doses taken within the 16-week period. This was analyzed as having a greater than 99% probability, that treatment acceptance and completion would exceed 80% under at least one of the three strategies.*

*The prompt addressing of curable TB is an important issue with clear benefits in mitigating death, especially amongst individuals with HIV. The 3HP randomized trial as described appears a significant contribution towards assessing treatment programmes in an African context. Additionally, the underlying raw data has also been provided as supplementary material. However, some reservations might be addressed.*

**Response**: We thank the reviewer for highlighting the importance of TB preventive therapy and the potential of the 3HP Options trial to make a significant contribution.

*1. The manuscript as it stands is fairly concise and focused. However, while the importance of the trial should not be understated, it is unclear as to whether an official publication on interim results is strictly warranted, especially as the trial is expected to complete by October 2022 (Line 103). It might be considered to support more strongly the case for publishing interim results (e.g. the trial qualifying for early stopping, in which case the analysis supporting early stopping might be presented; early stopping however does not appear to be catered for in the trial protocol, cited as [10])”*

**Response**: We appreciate the reviewer’s perspective and also struggled with the decision to pursue and publish an interim analysis. Although enrollment will be completed in October 2022, the last patients will not exit the 16-week treatment period until February 2023 – and it is likely that data cleaning, data analysis and manuscript writing will take a minimum of 6 additional months. Due to the strong ongoing interest in using 3HP to scale-up TB preventive therapy, the independent Trial Steering Committee unanimously recommended moving forward with the interim analysis. Based on this comment and similar comments below, we raised the issue again with the Trial Steering Committee (with an explicit statement that we would gladly accept a decision not to move forward with publication), and they remained unanimously in favor of publishing the interim analysis, on the basis of the urgency of these results – that waiting to publish these results until late 2023 may have an adverse impact on the global scale-up of 3HP for people living with HIV.

*2. More details or relevant citations on the Bayesian inference framework used (currently briefly described under the Statistical Analysis section from Line 134) for the main analysis, would be illuminating.*

**Response**: As requested, we have added in additional details in the Statistical Analysis section (lines 236-244) regarding the Bayesian analysis and a reference citing standard methods for ascertaining the posterior probability reported here:

“We used Bayesian inference to estimate the posterior probability that the proportion of PLHIV who accept and complete 3HP would exceed 0.8 under at least one of the three delivery strategies being compared (a co-primary trial hypothesis, as pre-specified in the trial protocol)*.* We used a beta distribution for the primary outcome and used a non-informative flat conjugate beta(1,1) prior. We conservatively assumed that outcomes were distributed equally across the three study arms; if outcomes were distributed unequally, the probability of the outcome proportion exceeding 0.8 would be higher in at least one arm. We then calculated the posterior probability that the proportion accepting and completing 3HP in a single arm exceeded 0.8 using standard methods taking advantage of the conjugate beta prior [14].”

*3. While Figure 2 contains primary outcome results stratified by subgroups, there does not appear analysis relating to delivery strategies (although existing results do indeed suggest that the various strategies likely result in treatment completion rates well in excess of 0.80). The Bayesian inference analysis framework might thus be explained in greater detail, and results also presented by treatment strategy (possibly also stratified by the existing subgroups in Figure 2)*

**Response**: Thank you for this comment. As noted, we do not report trial arm-specific outcomes as per the recommendation from our Trial Steering Committee (TSC). We raised the issue of presenting trial-arm specific outcomes again with the TSC in response to this comment, and they again recommended against reporting such data to maintain the integrity of the ongoing trial. We have included further details and a reference for the methods used to conduct the Bayesian inference analysis in the updated manuscript (lines 236-244; see response to Comment 2), and agree these results suggest the various strategies likely result in treatment completion rates well in excess of 0.80.

*4. Related to the above, the demographic characteristics in Table 1 might also be further stratified by treatment strategy, for completeness.*

**Response**: As noted in the response to the previous comment, our TSC recommended against reporting outcomes stratified by 3HP delivery strategy. Therefore, we did not modify Table 1 as suggested to keep the focus on the aggregate outcome analysis (and to maintain blinding of the study PIs).

***Reviewer #2:***

*This manuscript presents an interim analysis of outcome data aggregated across arms of the 3HP Options trial.  While the trial's primary objective is to compare different methods of delivering 3HP, the interim analysis presents treatment completion and adverse event outcomes for patients in all three arms together.  A very high proportion of patients accepted and completed treatment.  Given that this interim analysis does not address the question of how best 3HP can be delivered in a high-burden setting, its importance is framed as providing one of the first examples of 3HP being delivered in a programmatic setting in a high-burden country.  While I agree that publishing such examples is very important, I also think that the way the authors frame this report perhaps oversimplifies the situation; my concerns on this matter are described in comments 1 and 2 below.*

**Response**: We have tried to address the concerns in our responses to specific comments below.

*1) The argument that this publication reflects treatment completion achieved in a programmatic setting in a high-burden setting is key to the authors' justification of its importance.  However, this is a randomized trial being supported by the US NIH.  I realize that "programmatic setting" is not a strictly defined term and exists on a spectrum where research is embedded within programs.  But if the study paid for the digital adherence technology, staff monitoring treatment, or transport reimbursements, then it is hard to see how this is any more programmatic than the contexts of the 3HP efficacy trials, which had many sites in high-burden countries.  If the authors want to make this distinction between their trial and previous trials, then I think further description is necessary about what the study supported and how this is more programmatic than previous trials.*

**Response**: Thank you for this comment, and for the opportunity to clarify. We believe it is important to distinguish between trial design and execution, and the 3HP delivery strategies being evaluated in the trial. The design and execution of our trial is much more pragmatic than previous trials. Following consent and randomization, all aspects of 3HP treatment including drug administration, adherence monitoring, adverse event monitoring, and follow-up for missed doses or adverse events were performed by routine clinic staff – not by research staff funded by this study. Exclusion criteria were also intentionally kept to a minimum, reflecting only situations where 3HP would not be recommended in routine clinical practice. This is distinct from previous efficacy and effectiveness trials, wherein patients were carefully selected and closely monitored and followed-up by research staff throughout the course of treatment.

On the other hand, the 3HP delivery strategies intentionally differ from “usual care”, reflecting our belief that a comprehensive (yet feasible to implement) approach to 3HP delivery is necessary in order to achieve high levels of completion in routine practice. The components of the 3HP delivery strategies were chosen to target key barriers to 3HP acceptance and completion identified in formative research. The components were selected with input from patients and their health care providers to maximize acceptability and feasibility (*i.e.,* likelihood of scale-up if shown to be effective and cost-effective). Nonetheless, we recognize that similar strategies may not be taken up and therefore highlight in our conclusion that high levels of 3HP completion can be achieved in programmatic settings “particularly when delivery strategies are tailored to target known barriers to treatment completion”.

Based on the reviewer’s comment (and a similar comment made by Reviewer 5), we have further highlighted the pragmatic nature of the trial design and execution (lines 177-180, 188-190, 205-217, 308-310, 314-317).

*2) Lines 91-92: I agree that data on acceptance and completion of 3HP in high-burden programmatic settings is sparse. However, I am not sure it is so sparse as to warrant omitting references to any examples or data from these settings. China has been using a twice-weekly version of 3HP programmatically (experience is reviewed in Cui et al. Management of latent tuberculosis infection in China: exploring solutions suitable for high-burden countries; other papers have been published reporting data from specific centers or studies). A program in Pakistan has been using 3HP and published on its experience (Yuen et al. Cost of delivering 12-dose isoniazid and rifapentine versus 6 months isoniazid for tuberculosis infection in a high-burden setting). The iAdhere trial whose US-specific results are mentioned in the preivous sentence incidentally also included a site in South Africa.*

**Response**: Thank you for this comment. We have included the references you have kindly shared and further detailed the results of the iAdhere trial as further evidence for the need to generate additional evidence on 3HP acceptability and completion in settings such as ours:

“Notably, in the iAdhere trial, non-inferiority of SAT to DOT was only demonstrated among participating sites in the United States, with SAT performing worst (37% treatment completion) in South Africa, the only participating African site. These findings further underscore the need for evidence of 3HP acceptability and completion in high burden settings like Uganda.”

*3) Could the authors provide a sentence or two more to describe the type of digital adherence technology used? I realize that this may be in the previous protocol paper, but since the message of this paper is that supportive systems of delivering 3HP can achieve high completion rates in a high-burden setting, I think it is important to understand exactly what those supportive systems are.*

**Response**: Thank you for this suggestion. While full details of the 99DOTS-based platform are provided in the trial protocol paper, we have included some additional details here to describe its core features:

“The remaining doses were self-administered, and participants were asked to report dosing completion using the 99DOTS platform (Everwell Health Solutions, India). 99DOTS is a low-cost digital adherence technology wherein patients call random toll-free numbers pre-packaged with each medication dose and the calls are logged on the 99DOTS server to generate a real-time dosing history that health workers can monitor [15]. The 99DOTS platform was also used to send automated weekly IVR check-in calls asking “Are you well?” to assess for potential adverse events.”

*4) How was adverse event monitoring conducted? Verbal symptom screen? Was any routine laboratory monitoring performed?  What evaluations were performed if patients reported signs/symptoms of a potential adverse event?  I ask these questions because the secondary outcomes section reports the proportion of patients who had AE-related discontinuations, which is a very useful piece of information for programs. A description of AE monitoring would both help the reader assess the proportions being reported and also provide helpful information on how programs in high-burden settings can effectively monitor for AEs. Incidentally, if no routine laboratory monitoring was performed, it would be great if the authors could say this - one of the barriers frequently encountered to scaling up TB infection treatment in LMIC is the argument that the health system does not have the capacity for the type of LFT monitoring performed in high-income settings.*

**Response**: Thank you for this suggestion. Importantly, as described above in our response to Comment 1, AE monitoring was conducted by routine health care providers, not study staff trained in specific procedures. Briefly, pharmacy technicians used an adverse events checklist at all clinic visits (weekly for DOT; refill and end-of-treatment visits for SAT) to screen participants for potential side effects. In addition, pharmacy technicians were alerted when SAT participants responded “No” to the weekly IVR check-in (“Are you feeling well?”) and were asked to then call patients to administer the adverse events check list via the phone. Pharmacy technicians referred participants who reported any of the symptoms on the checklist or other concerning symptoms to a clinician for further evaluation. Any further assessment including laboratory testing was at the discretion of the clinician (*i.e.*, no routine laboratory monitoring was done during 3HP treatment). As requested, we have included additional details surrounding AE monitoring in Appendix 1 and under “Assessments and data collection” (lines 208-212):

“For adverse event monitoring, pharmacy technicians were asked to screen participants for potential side effects using a standardized checklist at clinic visits prior to 3HP dosing or by calling SAT participants who responded “No” to the weekly IVR check-in and to refer participants with potential side effects to a routine clinician for further evaluation. Further assessment including any laboratory testing was at the discretion of the clinician.”

*6) In the interest of making important information available to programs in high burden countries, it would be useful to know what the suspected 3HP-related adverse events were and their severity.*

**Response**: Thank you for this suggestion. We have included additional details about the suspected related AEs in the manuscript as well as in an Appendix submitted alongside our resubmission:

“Treatment was permanently discontinued due to a suspected 3HP-related adverse event in 8 (1.7%) participants (4 with pruritic rash, 1 with venous thromboembolism in the right leg, 1 with flu-like syndrome and peripheral neuropathy, 1 with pulmonary embolism, and 1 with acute liver injury – see Appendix 2 for additional details).”

*7) The discussion is rather abbreviated and does not delve much into discussing the supported self-administered mode of 3HP delivery, which I think is the most interesting aspect of this publication.  Ultimately, the study reports an expected finding of high 3HP completion, which is consistent with the evidence from other trials, the substantial literature published by programs in low-burden settings, and the limited programmatic experience published to date from high-burden settings.  To me, the value of this particular study is knowing how a program in a high-burden setting was able to achieve this high completion without DOT.  Discussing this experience and comparing it to other experiences with digital adherence technology, which have been something of a mixed bag in terms of real-world effectiveness, would add great value to the paper.*

**Response**: Thank you for these comments. We agree that the facilitated SAT strategy (including use of DAT) and the patient choice strategy (including use of shared decision making with a decision aid) are important and will likely be the most interesting aspects of this trial. However, we believe these issues are best highlighted in a future publication describing the full trial results. The full trial results will include a comparison of trial arm-specific outcomes whereas this interim analysis only includes outcomes aggregated across trial arms. Therefore, we do not believe it is appropriate to comment in the Discussion on one of the delivery strategies (such as SAT) when we do not present data on how it compares with the other delivery strategies being evaluated. That being said, we do agree that readers should be able to assess what was done in each arm – and more details are now provided in the Methods (lines 182-201) and full details are in the published protocol paper (manuscript document reference #14).

We respectfully disagree that high levels of 3HP completion in the context of routine care in a high burden setting is an expected finding. Outcomes from efficacy trials are often not replicated in real-world settings, and the implementation context is substantially different in low-burden vs high-burden settings. The high acceptance and completion of 3HP that have been observed in controlled clinical trials performed largely in low-burden/high-income settings merit confirmation when 3HP treatment is given in routine practice in high-burden/low-income settings (where the majority of people eligible for TB preventive therapy would be treated). Indeed, the Phase 3 iAdhere trial showed that completion rates were high at trial sites in high income countries but not at the one trial site in sub-Saharan Africa. Without such evidence, it is difficult to justify a global transition from isoniazid preventive therapy (which is cheaper) to 3HP. Thus, we believe there is value in demonstrating that high levels of 3HP completion can be achieved in the context of routine HIV/AIDS care with relatively low-cost delivery strategies that target known barriers to 3HP acceptance and completion. Based on the reviewer’s comments, we have expanded the Discussion to mention results of 3HP implementation in low-burden settings (lines 302-304):

“In low-burden and/or high-income countries, 3HP completion has been reported to be similar or higher in routine healthcare settings than rates reported in controlled clinical trials [16-19].”

***Reviewer #3:***

*The paper reports an interim analysis from a trial (3HP Options Trial) assessing the effect off three facilitated strategies of delivering 3HP regimen on treatment completion. The three study arms are facilitated DOT, facilitated SAT (including use of a digital adherence technology), and patient choice (with the assistance of a decision aid) between facilitated DOT and facilitated SAT. All participants receive automated dosing or clinic visit reminders weekly and are reimbursed transport costs for each clinic visit (11 visits for DOT & 2 for SAT).*

*The endpoint is treatment completion defined as accepting 3HP treatment and completing at least 11 of the 12 weekly doses within 16 weeks of enrolment. A Bayesian analysis was conducted to identify the posterior probability that at least one of the three arms will have treatment completion >80%. The analysis was based on participants randomised to 30 April 2021 with the target sample size expected to be completed in October 2022.*

*1.  The justification for this analysis was based on the urgent need for data in this in the context to aid in the  global 3HP scaleup, particularly in Uganda. It is not clear how this aligns with Uganda policy” on 3HP.*

**Response**: The Uganda Consolidated Guidelines for HIV prevention and treatment recently recommended 3HP as an alternative to INH. However, as far as we are aware, 3HP has not yet been rolled out at any HIV clinic in Uganda. There has been intense interest in our trial from the Ministry of Health and its local implementing partners. This was a key motivation for considering an interim analysis and was also cited by our Trial Steering Committee in recommending that we pursue publication of this interim analysis (see response to comment 5 below).

*2.  What is target sample size, expected to be completed in October 2022?*

**Response**: The target sample size for the full trial is 1,656 participants.

*3.  Is the trial design assessing for superiority or non-inferiority of study arms?*

**Response**: The trial design is assessing for superiority of study arms. Trial design details including sample size calculation considerations are included in the trial protocol (now included with our resubmission) and published trial protocol paper (reference #14 in the manuscript document).

*4.  It is not clear what it meant by hybrid type 3 effectiveness-implementation design*

**Response**: Thank you for the opportunity to clarify. We have now provided an explanation and relevant references under “Study design and participants” (lines 154-156):

“Hybrid effectiveness-implementation trials assess both effectiveness and implementation outcomes, with Type I designs having a greater focus on intervention effectiveness and Type 3 designs having a greater focus on implementation strategies and outcomes [9].”

*5.  How does this interim analysis relate to requests from external governance bodies such as a data safety monitoring board?*

**Response**: Thank you for this comment. Trial oversight is provided by an external Trial Steering Committee (TSC). The TSC is provided with a quarterly enrollment and adverse event report, which includes outcome data aggregated across arms, to facilitate trial monitoring**.** The issue of an interim publication was raised in the most recent TSC meeting. The TSC requested the investigators to prepare a document summarizing different options for an interim analysis/publication (including no interim analysis) and to list the pros and cons of each option. Following review and discussion of this document, the TSC unanimously recommended to proceed with an interim analysis and publication that was limited to outcome data aggregated across trial arms. This recommendation was reaffirmed after we shared the comments from reviewers of this paper, specifically those questioning whether an interim analysis is justified. We have clarified that the decision to report an interim analysis was supported by the TSC:

**“**The trial is registered on ClinicalTrials.gov (NCT03934931) and plans to report an interim analysis were reviewed and recommended by the independent Trial Steering Committee.”

*6.  It is not clear how treatment completion is assessed in the three arms  (doses taken at clinic visit, pill counts and /or DAT server data? ) - is the same method used across the three arms ?*

**Response**: Thank you for the opportunity to clarify. We have edited the language included under “Assessments and data collection” to more clearly indicate the methods for how 3HP initiation and completion is assessed, which is through pharmacy records and the 99DOTS server (lines 212-217, lines 223-224).

“Completion was assessed using the 99DOTS platform (i.e., weekly confirmation of self-administered doses by patient phone calls, with clinic-administered doses logged by pharmacy technicians on patients’ behalf). Self-administered doses were further verified by pharmacy technician pill count. Pharmacy technicians were asked to review participant dosing histories and call participants who missed clinic visits or did not call the 99DOTS platform to report expected self-administered doses.”

“To assess 3HP initiation and completion among participants in all three trial arms, research staff extracted weekly 3HP dosing data from the 99DOTS server.”

*7.  Why is outcome of "initiating 3HP and completing at least 11 of the 12 weekly doses within 16 weeks of enrolment chosen"?*

**Response**: The primary outcome of the trial reflects reach (proportion of individuals willing to initiate an intervention) and fidelity (completing the intervention as intended) of 3HP. The fidelity (i.e., treatment completion) component was defined in such a way as to be consistent with most previous trials of 3HP [11-13] and likely clinical implementation (i.e., providing patients some additional time and allowing for one missed dose – but requiring that >90% of all prescribed doses were taken).

*8.  How does the sample size in this interim analysis compare with the target sample size (expected to be completed in October 2022)?*

**Response**: The target sample size for the full trial is 1,656 participants, which was the number needed to power comparisons between trial arms. For this analysis, the sample size is 479.

*9.  The authors concluded that "Taken together, these results demonstrate high levels of 3HP uptake and completion can be achieved in a programmatic setting when delivery strategies are designed to address known barriers to TPT completion." Given the intervention arms have varying degrees of support and therefore varying costs attached, how does this issue contribute to faster roll-out of 3HP in the region. Would be interesting for the authors to comment on whether the costing of the interventions would also influence the type of intervention to be rolled-out? Which of the three interventions will the authors be recommending to the Ministry of Health in Uganda?*

**Response**: This interim analysis of trial data aims to disseminate preliminary and some of the first real-world data on 3HP completion in the context of routine care in a high-burden, programmatic setting. We believe these data support rapid scale-up of 3HP as an alternative to isoniazid preventive therapy (IPT), which has been plagued by low completion rates. Unfortunately, until the trial is completed, and we compare results between trial arms, we cannot recommend one of the 3HP delivery strategies over another. We are also collecting detailed costing data and will be able to compare the costs and cost-effectiveness of the 3 delivery strategies once the full trial is complete. Nevertheless, we believe that the scale-up of 3HP using one of the three strategies (based on programmatic preference) is justified by these results – and given that these results have been requested by Uganda Ministry of Health, we believe that they are likely to strongly influence policy related to 3HP scale-up in the country (and throughout the region).

*10. The authors report on the recently completed trial in South Africa, Ethiopia and Mozambique which found high levels of treatment completion achieved in a programmatic context. They continue by saying that their study highlights the value of facilitated delivery deliveries - though in reality we do not know the effects of the different interventions as the interim analysis combined data across the three arms.*

**Response**: Thank you for this comment. The reviewer is correct that we cannot say which of the three facilitated delivery strategies was the best because data were combined across arms. However, all three strategies included facilitation (streamlined clinic visits, transport reimbursement for clinic visits, dosing reminders). Regarding comparison to the WHIP3TB trial, we feel that those results may be questioned by the scientific community because completion was only assessed via pill count, and TB incidence was higher in the 3HP arm (with >90% completion) than in the isoniazid-only arm (with ~50% completion). As such, we believe it is reasonable to conclude that facilitation components that target key barriers were of value given the high treatment acceptance and completion rate across arms. However, the final trial analysis and results will be needed to determine which of the three strategies performed the best.

*11. It would be useful for the authors to expand on their assertion that this ongoing analysis helped inform time critical public health questions. Are the authors recommending this type of analysis for similar scenarios?*

**Response**: Thank you for the opportunity to clarify. This was a key aspect of our discussions with our Trial Steering Committee (TSC). The TSC members, all senior experts in HIV/TB care and global health, felt strongly that scale-up of TB preventive therapy among PLHIV was a key priority for global TB care and control, and that the interim trial data were highly relevant for informing country-level decisions about 3HP-based TB preventive therapy. Indeed, the Uganda Ministry of Health has requested the data reported here to inform their plans of whether to broadly scale-up 3HP to PLHIV. We do believe investigators should consider reporting interim analyses when they can inform time-sensitive public health questions, as has been done even for efficacy trials in the context of the COVID-19 pandemic [20-25], after carefully considering the potential impact of erroneous findings and impact on conduct of the remainder of the trial. This is even more true, we believe, for implementation trials where the intervention under evaluation has already been demonstrated to be efficacious. We have added text related to this to the Discussion:

“Further discussion is warranted among the scientific community as to when and how interim analyses of randomized trials – particularly trials focused on improving implementation of proven interventions – should be performed to inform public health decisions.”

***Reviewer #4:***

*This manuscript details an interim analysis of the ongoing 3HP Options Trial in Uganda, a randomized trial of DOT vs. SAT vs. SAT+DAT that is evaluating acceptance and completion rates across these three treatment strategies for TPT in PLHIV. Considering the need for more evidence for acceptance and adherence of these optimized strategies in the context of routine HIV care in sub-Saharan Africa, this manuscript will directly and substantially support the WHO's recommendation for using 3HP as an option for TPT in this setting.*

*The authors describe an inference analysis, estimating that at least one optimized treatment strategy would lead to >80% acceptance and completion of 3HP. Following their analysis, the authors demonstrated that all three strategies had a very high probability of exceeding 80% acceptance and treatment completion, effectively supporting that all three optimized strategies are highly effective in a sub-Saharan Africa context in Uganda.*

*The findings of this analysis are in context of previous literature on the topic, and the data analysis clearly supports the findings presented.*

*This manuscript details an interim analysis of an ongoing randomized trial and details of trial design have been previously published and are accessible via PubMed.*

*The overall study conforms to CONSORT guidelines for a randomized trial, and the methodology for the analysis to be reproduced.*

*Overall, the manuscript is well written, is accessible to non-specialists, and the findings will add to the body of evidence supporting high acceptability and completion rates for 3HP for TPT, using both DOT and SAT approaches, in the context of routine HIV care in sub-Saharan Africa.*

**Response**: We thank the reviewer for these comments. We have tried to respond to concerns raised in the comments below.

*1. Line 90 - consider emphasizing that the Belknap study demonstrated that SAT was noninferior to DOT, but only specifically in the United States component of this study (further supports the authors' argument that evidence on 3HP acceptability and completion rates from studies conducted outside the United States, especially in SSA, is needed.*

**Response**: Thank you for your suggestion, which we have incorporated in our edits to the introduction section (lines 142-145):

“Notably, in the iAdhere trial, non-inferiority of SAT to DOT was only demonstrated among participating sites in the United States, with SAT performing worst (37% treatment completion) in South Africa, the only participating African site. These findings further underscore the need for evidence of 3HP acceptability and completion in high burden settings like Uganda.”

*2. 114 - is the digital adherence technology (DAT) text based? Consider adding explanation on what the participant was required to do with DAT. IVR is clearly explained in the manuscript.*

**Response**: Sorry this was not clear. 99DOTS involves patients making toll-free phone calls to numbers pre-packaged with each medication dose. We have included these details in our revised manuscript (lines 191-198):

“For participants randomized to the facilitated SAT arm, the first, sixth and final 3HP doses were directly observed at clinic visits. The remaining doses were self-administered, and participants were asked to report dosing completion using the 99DOTS platform (Everwell Health Solutions, India). 99DOTS is a low-cost digital adherence technology wherein patients call random toll-free numbers pre-packaged with each medication dose and the calls are logged on the 99DOTS server to generate a real-time dosing history that health workers can monitor [15]. The 99DOTS platform was also used to send automated weekly IVR check-in calls asking “Are you well?” to assess for potential adverse events.”

*3. 129 - per the primary outcome: "the proportion of randomized participants who accept 129 3HP treatment and complete at least 11 of the 12 weekly doses within 16 weeks of enrollment." Did any participants who reached this outcome have a treatment interruption of 4 weeks before resuming weekly dosing and completing 3HP? I ask this as professional opinion can vary on the acceptable maximum treatment interruption allowed before a patient must restart 3HP (in my experience, between 1 and 4 weeks). Consider including the range of treatment interruption (in days) that was observed by this analysis for individuals who did successfully complete 3HP.*

**Response**: Thank you for this suggestion. We have included a sentence in the results detailing the largest treatment interruption among any patient completing treatment, which was 28 days (lines 272-274):

“The mean length of follow-up among those completing treatment was 78.4 days (standard deviation: 3.5 days) and ranged from 72 days to 109 days. The longest treatment interruption among any participant reaching the primary outcome was 28 days.”

*4. 150 (Figure 1) - one 'did not meet inclusion criteria' in figure 1 states: "resistance to isoniazid (INH) or rifapentine (RPT) (n=2)". How was this determined/known? Were these individuals previously treated for active TB disease and now qualified for TPT? Seems that would not be the case as another 'did not meet inclusion criteria' is "prior treatment for TB/preventive TB". Please clarify.*

**Response**: Thank you for pointing this out, which was an error we have now corrected. The two individuals included in the line referenced in the comment should have been included under “**contact with someone with INH- and/or rifampin-resistant TB”.** The updated figure is included in the resubmission on page 12.

*5. 179- considering describing most common adverse event(s) that led to 1.7% of participants discontinuing treatment.*

**Response**: Thank you for this suggestion. We have included additional details about the suspected related AEs in the manuscript as well as in an Appendix submitted alongside our resubmission:

“Treatment was permanently discontinued due to a suspected 3HP-related adverse event in 8 (1.7%) participants (4 with pruritic rash, 1 with venous thromboembolism in the right leg, 1 with flu-like syndrome and peripheral neuropathy, 1 with pulmonary embolism, and 1 with acute liver injury – see Appendix 2 for additional details). “

*6. 182 - unless it represents an increased risk for identifying the participant, consider including the name of the medication with a known 3HP interaction.*

**Response**: Thank you for your comment. We have edited the manuscript to include the names of the medications with drug-drug interactions with 3HP (lines 290-292).

“……1 (0.2%) had treatment discontinued due to starting new medications with known 3HP drug interaction (clarithromycin, omeprazole, domperidone), and 1 (0.2%) died in a motor vehicle accident.”

*7. The Discussion section does not include limitations. I encourage you to discuss some possible limitations of your analysis. One plausible limitation is that this is an interim analysis of 3HP uptake for an ongoing study in Kampala, Uganda, and the acceptability and treatment completion rates observed in this setting may not reflect the probability of acceptance and completion of 3HP in other countries and settings in sub-Saharan Africa.*

**Response**: Thank you for this suggestion – we have added a limitations section to the Discussion, including the limitation noted in your comment (lines 327-335):

“Our analysis has some limitations. First, we only assessed outcomes aggregated across trial arms; full trial results including comparison by arm will be presented at trial completion. Nevertheless, even when conservatively assuming that completion rates are similar across arms, our Bayesian analysis suggests a greater than 99% probability of exceeding 80% initiation and completion under at least one delivery strategy. Second, the trial was conducted at a single HIV/AIDS clinic in Uganda. However, the setting is similar to PEPFAR-funded HIV/AIDS clinics in other countries in sub-Saharan Africa, where similar barriers to completion of TB preventive therapy have been widely reported [1-8]. Third, some of the facilitation strategies evaluated as part of the 3HP Options trial are not likely to be part of usual care at most HIV/AIDS clinics in sub-Saharan Africa and would require additional funding to implement as part of 3HP scale-up.”

*8. Overall, a well written manuscript. My recommendation is 'accept with minor revision'.*

**Response**: We thank the reviewer for the positive assessment.

***Reviewer #5:***

*The authors present an interim analysis of limited data from the pragmatic randomized controlled trial, 3HP Options Trial, of the acceptance and completion (combined as a single outcome) of 3 months of weekly isoniazid and rifapentine (3HP) as TB preventive therapy (TBPT) in people living with HIV (PLHIV).  The 3HP Options Trial aims to compare the acceptance, completion and costs (among other outcomes) of 3 different 3HP delivery strategies. The trial is still enrolling and the outcome data from all three arms were combined and included as an aggregate for this analysis. There is no comparator group.*

*TBPT can prevent TB disease in PLHIV and practical alternatives to 6 months of daily isoniazid (which is standard of care in many regions) make an important contribution to public health. 3HP has been shown to be as effective as isoniazid for this purpose and is recommended by the WHO. As the authors note, uptake in previous clinical trials has varied between 38-50% and 90%. In this analysis, the authors report high acceptance and completion of 3HP as TBTP (92.9%), and calculate that this (i.e. acceptance and completion) would exceed 80% if one of the three treatment strategies in the trial were used in this setting. These results did not vary by sex, age or time on antiretroviral therapy.*

*The primary outcome/s of the 3HP Options Trial will hopefully indicate a best practice for dispensing 3HP to PLHIV in Uganda. Other elements of the study design may provide a template for stakeholder and community engagement and support patient-centred care. The aggregation of the data in these analyses limits the novelty and usefulness of the results. In addition, as presented, the description of the settings and methods do not support the conclusion that the results are generalizable to a programmatic setting.*

*The manuscript is very well-written however, the impact is overstated. It may be better suited to an alternative publication. Given the remit that 'papers published in PLOS Medicine should be of the highest technical quality and be seen as significant advances within their own discipline and beyond' I do not think this interim analysis is suitable for publication in this journal.*

**Response**: We have tried to address your concern about the potential impact of this interim analysis in our responses to specific comments below.

*Major comments*

*1. While the 3HP Options Trial may be implemented in a routine clinical setting, the trial study procedures are not routine and vary from intensive observed monitoring (with re-imbursement for visits) to digital adherence monitoring and automated dosing and clinic reminders. The implication that the results have been achieved in a programmatic context (line 198) is that they are generalizable to similar routine clinical settings in Uganda and elsewhere in sub-Saharan Africa. Such practices described above are not routine in most programs which are often under-resourced.*

**Response**: This comment is similar to Comment 1 made by Reviewer 2. We have copied our response to that comment here as well:

“Thank you for this comment, and for the opportunity to clarify. We believe it is important to distinguish between trial design and execution, and the 3HP delivery strategies being evaluated in the trial. The design and execution of our trial is much more pragmatic than previous trials. Following consent and randomization, all aspects of 3HP treatment including drug administration, adherence monitoring, adverse event monitoring, and follow-up for missed doses or adverse events were performed by routine clinic staff – not by research staff funded by this study. Exclusion criteria were also intentionally kept to a minimum, reflecting only situations where 3HP would not be recommended in routine clinical practice. This is distinct from previous efficacy and effectiveness trials, wherein patients were carefully selected and closely monitored and followed-up by research staff throughout the course of treatment.

On the other hand, the 3HP delivery strategies intentionally differ from “usual care”, reflecting our belief that a comprehensive (yet feasible to implement) approach to 3HP delivery is necessary in order to achieve high levels of completion in routine practice. The components of the 3HP delivery strategies were chosen to target key barriers to 3HP acceptance and completion identified in formative research. The components were selected with input from patients and their health care providers to maximize acceptability and feasibility (*i.e.,* likelihood of scale-up if shown to be effective and cost-effective). Nonetheless, we recognize that similar strategies may not be taken up and therefore highlight in our conclusion that high levels of 3HP completion can be achieved in programmatic settings “particularly when delivery strategies are tailored to target known barriers to treatment completion”.

Based on the reviewer’s comment (and a similar comment made by Reviewer 5), we have further highlighted the pragmatic nature of the trial design and execution (lines 177-180, 188-190, 205-217, 308-310, 314-317).”

As stated in that response, we have revised our conclusion to highlight that high levels of 3HP completion can be achieved in programmatic settings “particularly when delivery strategies are tailored to target known barriers to treatment completion”. We do not wish to imply that, simply because results are achievable in a programmatic context, they can necessarily be replicated in all resource-limited settings. Ultimately, we believe programs will need to support not just costs of new technologies (drugs, diagnostics etc.) but the supportive measures needed to implement them effectively. The facilitation strategies used in the trial were selected with input of local stakeholders with scale-up feasibility a key consideration.

*2. The authors reference the 3HP Options Trial protocol but do not describe the setting, including physical, technological and human resources. Did clinic staff receive incentives for participating in the trial management? What was the source of funds for re-imbursement? At what level was the political and management support provided? Related to the lack of detail in the methodology, streamlined clinic visits and other patient-centred (line 202) and facilitated delivery strategies (line 186) are not described but are alluded to in the discussion. In addition, one of the strengths of the study design is noted to be the engagement of relevant stakeholders (210), but this does not appear elsewhere in the manuscript (methods or results).*

**Response**: Thank you for this comment. Under the Study setting section we have added the following details regarding the Mulago ISS clinic and its leadership:

“The trial is being conducted among adults accessing HIV/AIDS care at the Mulago Immune Suppression Syndrome (ISS; i.e., HIV/AIDS) clinic in Kampala, Uganda. This clinic is the largest outpatient HIV clinic in Uganda (over 16,000 PLHIV enrolled and 300 new PLHIV registered monthly), and scale-up of TB preventive therapy is a key priority for clinic leadership.”

Under Assessments and Data collection, we have clarified that existing clinic staff provided 3HP-related care and did not receive any incentives:

“Clinic staff did not receive any incentives or payments for delivering care related to 3HP.”

Under “Randomization and Interventions”, we have added the following related to stakeholder engagement:

“The delivery strategies were designed to address barriers to 3HP acceptance and completion identified in formative research [26-28] and with involvement of local stakeholders including PLHIV, clinicians, and clinic/program leadership to maximize acceptability and feasibility.”

We have also added more details on the 3HP delivery strategies in this section.

Last, because this trial is seeking to identify the optimal delivery strategy for 3HP, it paid for some components of the delivery strategies including 99DOTS set up and maintenance and transport reimbursements. However, these components were selected in consultation with clinic and program leadership as aspects that could be delivered by the clinic/program at scale if shown to be effective. We have also clarified that the level of transport reimbursement was selected based on an assessment of local transport costs as well as what was considered to be acceptable at scale by clinic/program leadership.

*Minor comments*

*1. Is the trial registered?*

**Response**: Trial registration details are now included in the Methods section (line 172).

*2. Study design: The power calculations have been based on the parent study which aims to enrol 1656 adults. Approximately 30% of the target have been enrolled. Is this sufficient for the interim analysis to be meaningful?*

**Response**: The sample size and power estimates for the full trial are based on making comparisons between trial arms, which we do not do in this interim analysis. Instead, we report outcome data aggregated across trial arms. We used Bayesian inference analysis to determine the probability that the outcome proportion is >80% in at least one arm, given the observed aggregate outcome proportion. This posterior probability was >99.9%, indicating there was adequate power for this analysis.

*3. As noted above, the background planning and the integration of the study procedures into the routine setting are not well described. This is necessary if the conclusion drawn is that the systems work in a program setting.*

**Response**: Many of these details can be found in our trial protocol paper; however, in response to this comment, we have taken the opportunity to clarify in the manuscript the routine care channels through which 3HP treatment was delivered as part of our pragmatic trial (lines 188-190, 205-217, 308-310).

**REFERENCES**

1.	Getahun H, Granich R, Sculier D, Gunneberg C, Blanc L, Nunn P, et al. Implementation of isoniazid preventive therapy for people living with HIV worldwide: barriers and solutions. AIDS. 2010;24 Suppl 5:S57-65.

2.	Jacobson KB, Niccolai L, Mtungwa N, Moll AP, Shenoi SV. "It's about my life": facilitators of and barriers to isoniazid preventive therapy completion among people living with HIV in rural South Africa. AIDS Care. 2017;29(7):936-42.

3.	Rowe KA, Makhubele B, Hargreaves JR, Porter JD, Hausler HP, Pronyk PM. Adherence to TB preventive therapy for HIV-positive patients in rural South Africa: implications for antiretroviral delivery in resource-poor settings? Int J Tuberc Lung Dis. 2005;9(3):263-9.

4.	Mindachew M, Deribew A, Memiah P, Biadgilign S. Perceived barriers to the implementation of Isoniazid preventive therapy for people living with HIV in resource constrained settings: a qualitative study. Pan Afr Med J. 2014;17:26.

5.	Kim HY, Hanrahan CF, Dowdy DW, Martinson NA, Golub JE, Bridges JFP. Priorities among HIV-positive individuals for tuberculosis preventive therapies. Int J Tuberc Lung Dis. 2020;24(4):396-402.

6.	Cowan J, Greenberg Cowan J, Barnhart S, Demamu S, Fiseha D, Graham W, et al. A qualitative assessment of challenges to tuberculosis management and prevention in Northern Ethiopia. Int J Tuberc Lung Dis. 2013;17(8):1071-5.

7.	Gust DA, Mosimaneotsile B, Mathebula U, Chingapane B, Gaul Z, Pals SL, et al. Risk factors for non-adherence and loss to follow-up in a three-year clinical trial in Botswana. PLoS One. 2011;6(4):e18435.

8.	Makanjuola T, Taddese HB, Booth A. Factors associated with adherence to treatment with isoniazid for the prevention of tuberculosis amongst people living with HIV/AIDS: a systematic review of qualitative data. PLoS One. 2014;9(2):e87166.

9.	Brown CH, Curran G, Palinkas LA, Aarons GA, Wells KB, Jones L, et al. An Overview of Research and Evaluation Designs for Dissemination and Implementation. Annu Rev Public Health. 2017;38:1-22.

10.	Glasgow RE, Vogt TM, Boles SM. Evaluating the public health impact of health promotion interventions: the RE-AIM framework. Am J Public Health. 1999;89(9):1322-7.

11.	Belknap R, Holland D, Feng PJ, Millet JP, Cayla JA, Martinson NA, et al. Self-administered Versus Directly Observed Once-Weekly Isoniazid and Rifapentine Treatment of Latent Tuberculosis Infection: A Randomized Trial. Ann Intern Med. 2017;167(10):689-97.

12.	Sterling TR, Villarino ME, Borisov AS, Shang N, Gordin F, Bliven-Sizemore E, et al. Three months of rifapentine and isoniazid for latent tuberculosis infection. N Engl J Med. 2011;365(23):2155-66.

13.	Moro RN, Borisov AS, Saukkonen J, Khan A, Sterling TR, Villarino ME, et al. Factors Associated With Noncompletion of Latent Tuberculosis Infection Treatment: Experience From the PREVENT TB Trial in the United States and Canada. Clin Infect Dis. 2016;62(11):1390-400.

14.	Gelman A, Carlin JB, Stern HS, Dunson DB, Vehtari A, DB R. Bayesian data analysis. Boca Raton: CRC Press; 2014.

15.	Subbaraman R, de Mondesert L, Musiimenta A, Pai M, Mayer KH, Thomas BE, et al. Digital adherence technologies for the management of tuberculosis therapy: mapping the landscape and research priorities. BMJ Glob Health. 2018;3(5):e001018.

16.	Sandul AL, Nwana N, Holcombe JM, Lobato MN, Marks S, Webb R, et al. High Rate of Treatment Completion in Program Settings With 12-Dose Weekly Isoniazid and Rifapentine for Latent Mycobacterium tuberculosis Infection. Clin Infect Dis. 2017;65(7):1085-93.

17.	Walker RE, Bass S, Srinivas P, Miranda C, Johnson L, Pallotta AM. Evaluation of 3 Months of Once-Weekly Rifapentine and Isoniazid for Latent Tuberculosis Infection. Ann Pharmacother. 2020;54(5):457-63.

18.	Sun HY, Huang YW, Huang WC, Chang LY, Chan PC, Chuang YC, et al. Twelve-dose weekly rifapentine plus isoniazid for latent tuberculosis infection: A multicentre randomised controlled trial in Taiwan. Tuberculosis (Edinb). 2018;111:121-6.

19.	Haas MK, Aiona K, Erlandson KM, Belknap RW. Higher Completion Rates with Self-administered Once-weekly Isoniazid-Rifapentine versus Daily Rifampin in Adults with Latent Tuberculosis. Clin Infect Dis. 2020.

20.	Xia S, Duan K, Zhang Y, Zhao D, Zhang H, Xie Z, et al. Effect of an Inactivated Vaccine Against SARS-CoV-2 on Safety and Immunogenicity Outcomes: Interim Analysis of 2 Randomized Clinical Trials. JAMA. 2020;324(10):951-60.

21.	Logunov DY, Dolzhikova IV, Shcheblyakov DV, Tukhvatulin AI, Zubkova OV, Dzharullaeva AS, et al. Safety and efficacy of an rAd26 and rAd5 vector-based heterologous prime-boost COVID-19 vaccine: an interim analysis of a randomised controlled phase 3 trial in Russia. Lancet. 2021;397(10275):671-81.

22.	Voysey M, Clemens SAC, Madhi SA, Weckx LY, Folegatti PM, Aley PK, et al. Safety and efficacy of the ChAdOx1 nCoV-19 vaccine (AZD1222) against SARS-CoV-2: an interim analysis of four randomised controlled trials in Brazil, South Africa, and the UK. Lancet. 2021;397(10269):99-111.

23.	Baden LR, El Sahly HM, Essink B, Kotloff K, Frey S, Novak R, et al. Efficacy and Safety of the mRNA-1273 SARS-CoV-2 Vaccine. N Engl J Med. 2021;384(5):403-16.

24.	Jackson LA, Anderson EJ, Rouphael NG, Roberts PC, Makhene M, Coler RN, et al. An mRNA Vaccine against SARS-CoV-2 - Preliminary Report. N Engl J Med. 2020;383(20):1920-31.

25.	Anderson EJ, Rouphael NG, Widge AT, Jackson LA, Roberts PC, Makhene M, et al. Safety and Immunogenicity of SARS-CoV-2 mRNA-1273 Vaccine in Older Adults. N Engl J Med. 2020;383(25):2427-38.

26.	Kadota JL, Katamba A, Musinguzi A, Welishe F, Nabunje J, Ssemata JL, et al. Willingness to accept reimbursement for visits to an HIV clinic for tuberculosis preventive therapy. Int J Tuberc Lung Dis. 2020;24(7):729-31.

27.	Lim RK, Semitala FC, Atuhumuza E, Sabiti L, Namakula-Katende J, Muyindike WR, et al. Patient choice improves self-efficacy and intention to complete tuberculosis preventive therapy in a routine HIV program setting in Uganda. PLoS One. 2021;16(2):e0246113.

28.	Semitala FC, Musinguzi A, Ssemata JL, Welishe F, Nabunje J, Kadota JL, et al. Acceptance and completion of rifapentine based TB preventive therapy (3HP) among people living with HIV (PLHIV) in Kampala, Uganda—patient and health worker perspectives. Implementation Science Communications. 2021;2.
