# Supplementary material for: Completion of isoniazid–rifapentine (3HP) for tuberculosis prevention among people living with HIV: Interim analysis of a hybrid type 3 effectiveness–implementation randomized trial
Source: PLoS Med. 2021 Dec 16;18(12):e1003875. doi: 10.1371/journal.pmed.1003875 (PMC8726462; doi:10.1371/journal.pmed.1003875)
Supplement: Attachment — Submitted filename: 3HP Options Trial_Interim Analysis_2nd Review response.docx [file pmed.1003875.s010.docx]

Dear Dr. Davidson,

Thank you for your additional comments on our manuscript titled "Completion of Isoniazid-Rifapentine (3HP) for tuberculosis prevention among people living with HIV: interim analysis of a hybrid type 3 effectiveness-implementation randomized trial", and for the welcome news that the paper will be accepted for publication*.* We have made the additional edits to the manuscript requested from the editors and the reviewers, which we have outlined below. We look forward to its publication in *PLOS Medicine.*

**Requests from Editors:**

*1. Please remove the abbreviations "TB" and "PLHIV" from the title.*

**Response:** Thank you, these abbreviations have been removed.

*2. Please include the baseline summary data (from lines 251-253) in your abstract.*

**Response:** Thank you for this suggestion. We have included the following sentence in the abstract: “Of these 479 participants, 309 (65%) were women, mean age was 41.9 years (standard deviation (SD): 9.2), and mean time on antiretroviral therapy (ART) was 7.8 years (SD: 4.3).”

*3. Line 119: Please state the number of patients discontinued (8) as well as the percentage.*

**Response:** The number is now reflected in addition to the percentage.

*4. Please define AST and ALT in Figure 1.*

**Response:** The edited figure is included on page 12 of the resubmitted manuscript.

*5. Thank you for including a STARI checklist. Please adapt the checklist to use sections and paragraph numbers rather than page numbers (which will likely change during the revision process). Column headers can be updated to reflect this change (from 'page number' to 'Section name and paragraph number', or similar).*

**Response:** The updated version of the STARI checklist including the section names and paragraph numbers is included with our resubmission.

*6. To help us extend the reach of your research, please provide any Twitter handle(s) that would be appropriate to tag, including your own, your co-authors, your institution, funder, or lab.*

**Response:**

Adithya Cattamanchi: @acattamanchi

David Dowdy: @davidwdowdy

UCSF PRISE Center: @ImSatUCSF

**Comments from Reviewers:**

*Reviewer #1: We thank the authors for acknowledging our prior concerns. The main reservation remains that this is an interim analysis with arm-specific details unable to be further provided to maintain the integrity of the ongoing trial. That said, the primary objective (i.e. a greater than 99% probability that treatment acceptance and completion would exceed 80%, under at least one of the three strategies) appear highly likely to have been attained, and the release of these interim results (as opposed to late 2023 for the full results) would appear to help guide 3HP treatment promptly. Considering this, we have in principle no objection to the release of these results if judged significant enough by the editors, particularly if the inability to reveal arm-stratified analysis is further emphasized as a limitation.*

**Response:** Thank you for your careful review and for the favorable recommendation. We will further highlight the limitation as advised.

*Reviewer #2: Thank you to the authors for the revision. The paper is substantially more balanced in the way it contextualizes its significance, and it is more informative for programs considering how to deliver 3HP in high-burden settings.  I agree with the other reviewers who have questioned the importance of an interim results publication that neither answers the question of how best 3HP can be delivered nor discusses the implementation experience with these methods (presumably saving both for what will be the "main" trial publication).  However, I think that on the balance, the information that is now presented about the use of 99DOTS, adverse event monitoring under programmatic conditions, and the use of transport reimbursements and other patient support strategies, in the context of overall high treatment completion, gives sufficient value to warrant more prompt dissemination.

The one comment I have is the added citation of the RE-AIM framework.  I am sure that the larger study was indeed designed using the RE-AIM framework, but it is difficult to see how this interim report maps to it.  It is not clear from the description of the trial that 3HP is the intervention and DOT/99DOTS are the implementation strategies, so it is hard to intuitively see treatment completion as a fidelity measure since it is often treated as a proxy effectiveness measure.  In addition, it is confusing to cite use of the RE-AIM framework when only two of the components are addressed as a composite outcome, and the other three are not addressed at all (adoption and maintenance are not even mentioned among the outcomes referenced in the protocol).  I suggest clarifying in the study design section that 3HP is the evidence-based intervention and that DOT vs supported SAT are the implementation strategies being assessed.  And I suggest either briefly summarizing the main outcomes corresponding to the RE-AIM components or at the very least being explicit that this interim analysis addresses only a combined reach+implementation fidelity outcome, while the other RE-AIM components will be more fully assessed at the end of the trial.*

**Response:** Thank you for the favorable review, and for your suggestions and opportunity to clarify. As suggested, we have clarified in the study design section that 3HP is the evidence-based intervention and that facilitated DOT, facilitated SAT and patient choice are the implementation strategies being assessed:

*“*Our overall goal as a Type 3 hybrid design is to identify the optimal implementation strategy (facilitated DOT, facilitated SAT, or patient choice) for enabling completion of 3HP (*i.e.,* the evidence-based intervention), while also adding to the large body of evidence demonstrating its safety and effectiveness*.”*

Also as suggested, we have clarified that this interim analysis addresses only a combined reach+implementation fidelity outcome, while the other RE-AIM components will be reported at the end of the trial:

“Trial outcomes were developed using the RE-AIM evaluation framework [19]. The primary outcome is the proportion of randomized participants who accept 3HP treatment and complete at least 11 of the 12 weekly doses within 16 weeks of enrollment [7, 9, 10], which reflects intervention reach (acceptance) and fidelity (treatment completion). In addition, this interim analysis assessed the proportion of participants initiating 3HP treatment who had a serious adverse event (AE), defined as any AE resulting in treatment discontinuation, which reflects the effectiveness domain of the RE-AIM framework. Additional trial effectiveness and implementation outcomes are described in the published trial protocol [14] and will be reported upon completion of the trial.”

*Reviewer #3: Thank you for your detailed response to comments. My concerns have been addressed.*

**Response:** Thank you for the opportunity to address your comments/concerns.

*Reviewer #4: I very much agree with Reviewer #5's comment that this interim research, overall, may not meet full PLOS Medicine criteria for publication, which includes the language that publications "be seen as significant advances within their own discipline and beyond". As an interim analysis of 3HP TPT acceptance and completion, based on aggregated data across the study arms being evaluated, this manuscript may not fully fit PLOS Medicine criteria for publication. Though this interim analysis does not address the question of how best 3HP can be delivered in a high-burden setting (the authors do state the final research will address this), it does, importantly, provide one of the first examples of 3HP being delivered in a programmatic setting in a high-burden country, demonstrating positive acceptance and fidelity of 3HP, in general. For the latter findings, I recommend this manuscript be accepted for publication, but respectfully defer to the Editor's final decision on if this manuscript represents a significant advance in the field of TPT and meets journal criteria for publication.*

**Response:** Thank you for your careful review and for the favorable recommendation.

*Reviewer #5: Much more detail and clarity has been provided and the manuscript improved. The tone has been moderated and the data presented more appropriately (to my mind); my comments have been addressed. The manuscript is sound and well-written.*

*I am still not certain that the impact is sufficient for publication in PLOS MEDICINE, but the editors are better placed in this regard.*

**Response:** Thank you, we are glad we sufficiently addressed your concerns and appreciate your close review of our manuscript.
